# A snapshot of Late Mesolithic life through death: An appraisal of the lithic and osseous grave goods from the Castelnovian burial of Mondeval de Sora (Dolomites, Italy)

**DOI:** 10.1371/journal.pone.0237573

**Published:** 2020-08-14

**Authors:** Federica Fontana, Emanuela Cristiani, Stefano Bertola, François Briois, Antonio Guerreschi, Sara Ziggiotti

**Affiliations:** 1 Dipartimento di Studi Umanistici, Università degli Studi di Ferrara, Ferrara, Italy; 2 Department of Oral and Maxillo-Facial Sciences – DANTE - Diet and ANcient TEchnology Laboratory, Sapienza University of Rome, Rome, Italy; 3 Laboratoire TRACES – UMR 5608 Université Jean Jaurès, Maison de la Recherche, Toulouse, France; 4 Dipartimento di Territorio e Sistemi Agro-Forestali, Università degli Studi di Padova, Legnaro, Padova, Italy; University at Buffalo - The State University of New York, UNITED STATES

## Abstract

The Late Mesolithic in Southern Europe is dated to the 7th and the first part of the 6th millennia BCE and is marked by profound changes which are mostly evident in the technical know-how and tool-kit of the last hunter-fisher-gatherer societies. The significance of this phase also relates to the fact that it precedes the Early Neolithic, another period of major transformations of human societies. Nonetheless, the Late Mesolithic still remains a poorly known age in this area. A burial discovered at Mondeval de Sora (Northern Italy) in 1987, represents a unique window into this period. In this paper, we provide a detailed analysis of more than 50 lithic and osseous artifacts associated with this burial. We highlight important contextual data regarding the techno-economic dimension and the notion of personal burial possessions. Based on the association and location of some items, we propose a new interpretation of the social status of this individual and the possible impact of technological innovation on the social organization and symbolic sphere of Late Mesolithic groups.

## Introduction

The Late Mesolithic in Southern Europe spans the epoch of the 7th and the first centuries of the 6th millennia BCE [1]. With respect to the previous phase of the Mesolithic, this period is signed by profound changes, which had a particular impact on the tool-kit of the last hunter-fisher-gatherer societies [1–4]. While we know that the new traditions–mostly represented by the introduction of more sophisticated flint-knapping techniques that allowed producing regular blades and bladelets from which new categories of armatures and tools were obtained—spread rapidly across most regions of the continent, their origin and modality of diffusion are still debated. Two main hypotheses have proposed either a Northern African or an Eastern Eurasian origin, both regions attesting an earlier appearance of such new technical know-how and material culture repertoire than Southern Europe. Yet, no definite evidence is so far available to confirm any of the two [4–8]. The significance of Late Mesolithic also relates to the fact that it precedes the Early Neolithic. Therefore, reconstructing the lifeways of these populations is also crucial in order to understand the impact of the Neolithization process in the different regions of the continent. This was a complex phenomenon, which involved the interaction between people arriving from the Near East and the local European populations. Such interactions may have varied considerably from region to region, and, in several cases, they remain poorly known also due to a lack of information about the Late Mesolithic occupations of several areas and the scarcity of radiometric dating [9–11].

This paper focuses on the sole Late Mesolithic burial documented in northern Italy, where this period is represented by the so-called Castelnovian complex, a culture-historical and typological label defined in the 1960s [12] and geographically extending from the low Rhone valley in France to western Slovenia. At a larger scale, such cultural facies may be considered as part of the Blade and Trapezes Complex [2] as it presents several affinities with the Geometric Mesolithic industries from Iberia [13], the Late Mesolithic from the Eastern Adriatic region [14], and the Castelnovian from Sicily [15]. Although a quite large Castelnovian record is attested across Northern Italy, our understanding of this phase of prehistory in this region still remains poor. Evidence is mostly represented by find-spots, while only a few sites have been the object of extensive investigations, which have either yielded lithic assemblages only or, have been explored over small surfaces mainly. Overall, this Castelnovian record shows a similar distribution pattern to that of the Early Mesolithic Sauveterrian culture complex, which reflects an intense occupation of both mountainous (central and eastern Alps, Emilian Apennines) and plain areas (Po and interconnected alluvial plains) [16–20]. Consequently, the decline in the occupation intensity of upland territories with a shift towards pre-Alpine and plain areas during the Castelnovian, originally proposed in the 1980s [21,22], has now been called into question by the evidence brought to light in several areas of the central and eastern-southern Alps [16,20,23]. Further confirmation of this hypothesis is represented by the burial found at Mondeval de Sora, which represents the focus of this paper. Consequently, it is argued that the shifts in the settlement patterns of Late Mesolithic groups in this area are indicative not necessarily of a decline in the occupation of upland, mountainous zones, but rather of a change in the pattern of mobility towards an increased logistical behavior with respect to the early Mesolithic [24].

As regards to material culture traditions, changes in lithic assemblages observed within the Castelnovian are shared across a wide area of the Mediterranean as well as along the Atlantic coasts of Western Europe [1,4,25]. The changes are evidenced in the production of regular blades and bladelets obtained by the adoption of two new knapping techniques: pressure and indirect percussion. The use of each of these techniques has been ascertained so far in a few sites of this region so that a detailed map of their presence and chronology is still not available [26,27]. The appearance of a new type of arrowhead with a trapezoidal shape is also attested along with that of laterally notched bladelets [28,29]. Technological innovations also characterize Castelnovian osseous technology, as a new type of tool–harpoon–became an important component of the tool-kit of Alpine hunter-gatherers-fishers during this period [29,56].

While the general patterns of technological changes are known, data on the Castelnovian socio-economical strategies are still scanty as well as those on symbolic behavior [3,6,9,16,20]. In particular, some items fashioned into various forms of “art” discovered at Riparo Gaban in the Adige Valley (NE Italy), such as a female sculpture made out of a red deer antler, represent unique objects [30,31]. Furthermore, items used for personal adornment, mostly perforated shells and red deer atrophic canines, have been found at different sites attesting to a continuity of the Mesolithic decorative tradition from the earlier Sauveterrian period onwards [32].

With regard to funerary rites, the burial at Mondeval de Sora represents the sole context known for the entire Castelnovian complex of Northern Italy and one of the few Late Mesolithic examples in southern Europe. This paper discusses the results of a recent re-analysis of the rich collection of lithic and osseous items, all remarkably preserved, which forms the greatest part of the Mondeval de Sora grave assemblage [24,33,34]. This new analysis includes the characterization of raw materials, the investigation of manufacturing techniques and the identification of use wear traces on both of the above-mentioned categories of implements.

All specimens described in the manuscript were studied and photographed under permits granted by the Soprintendenza Archeologia del Veneto and Museo “Vittorino Cazzetta”.

Given the rarity of several types of objects represented in the funerary assemblage, this study provides a unique snapshot of the technological, socio-economical, and ritual habits of Late Mesolithic societies, as well as of the personal identity traits of the deceased.

### The Castelnovian burial of Mondeval de Sora

**Fig 1:**
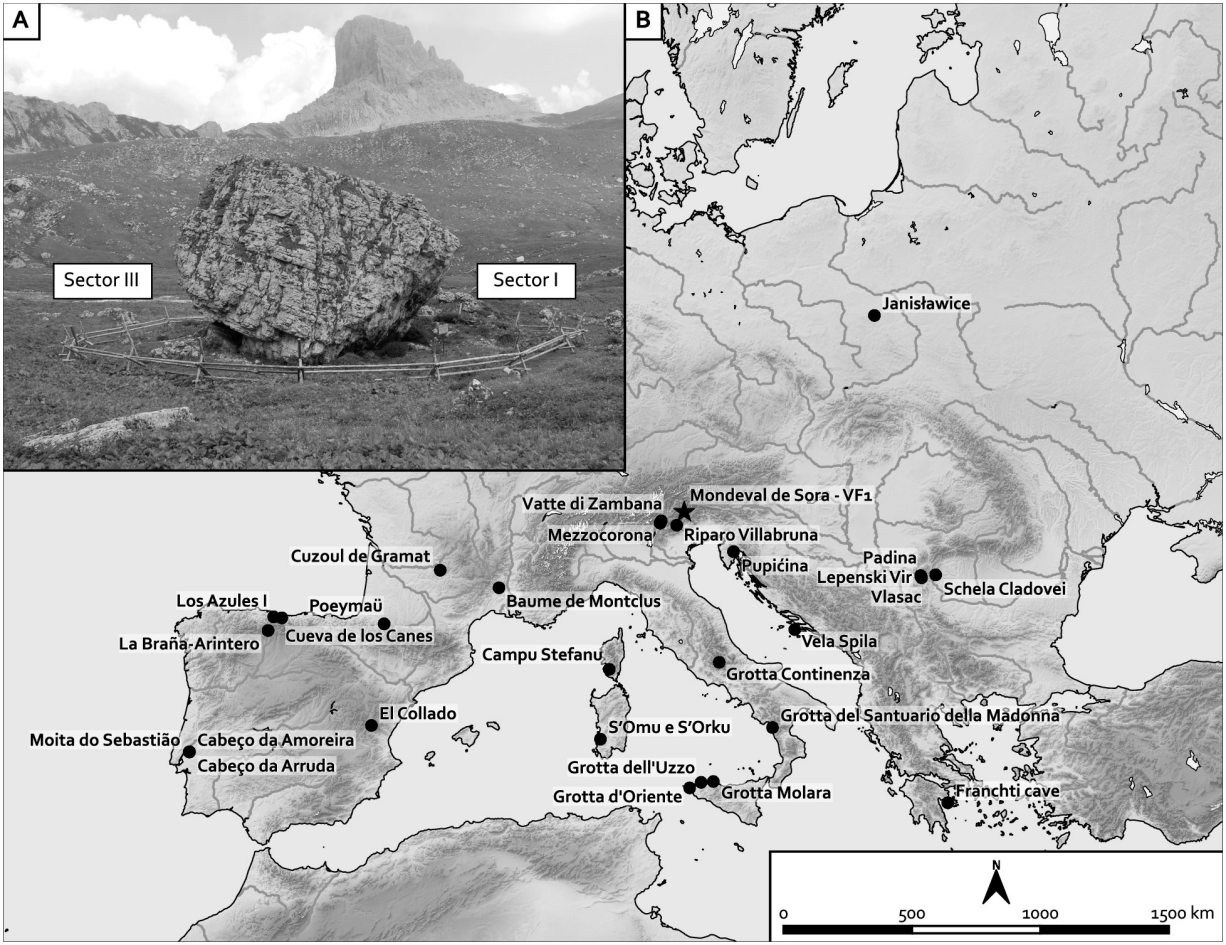
The site of Mondeval de Sora with Sector I, on the right, and Sector III, on the left (photo: A. Guerreschi) (A); location of the site of Mondeval de Sora and other main sites with Mesolithic burial grounds in Southern and Central Europe cited in the text, and the Late Epigravettian site of Riparo Villabruna (B).

The site of Mondeval de Sora (Val Fiorentina 1, San Vito di Cadore, N-E Italy, 12.09397°E, 46.46666°N -WGS84) is located under the overhang of a large erratic mass on a terrace lying 2,150 m.a.s.l. in the high valley of the Piave river (Belluno Dolomites, south-eastern Alps). Fieldwork at the site was carried out annually between 1986 and 2000, revealing traces of human occupation under three sides of the boulder (Sectors I, II, III) (Fig 1A). The Castelnovian burial was identified and excavated in Sector I. The stratigraphy of Sector I spanned from the Early Mesolithic to the Medieval age. Mesolithic levels were preserved solely in the southern portion of this Sector and were mostly represented by Early Mesolithic (Sauveterrian, Late Preboreal/early Boreal) dwelling structures and anthropic layers. The very limited preserved portions of layers referred to the Castelnovian occupation are still unpublished [33,35–39].

**Fig 2:**
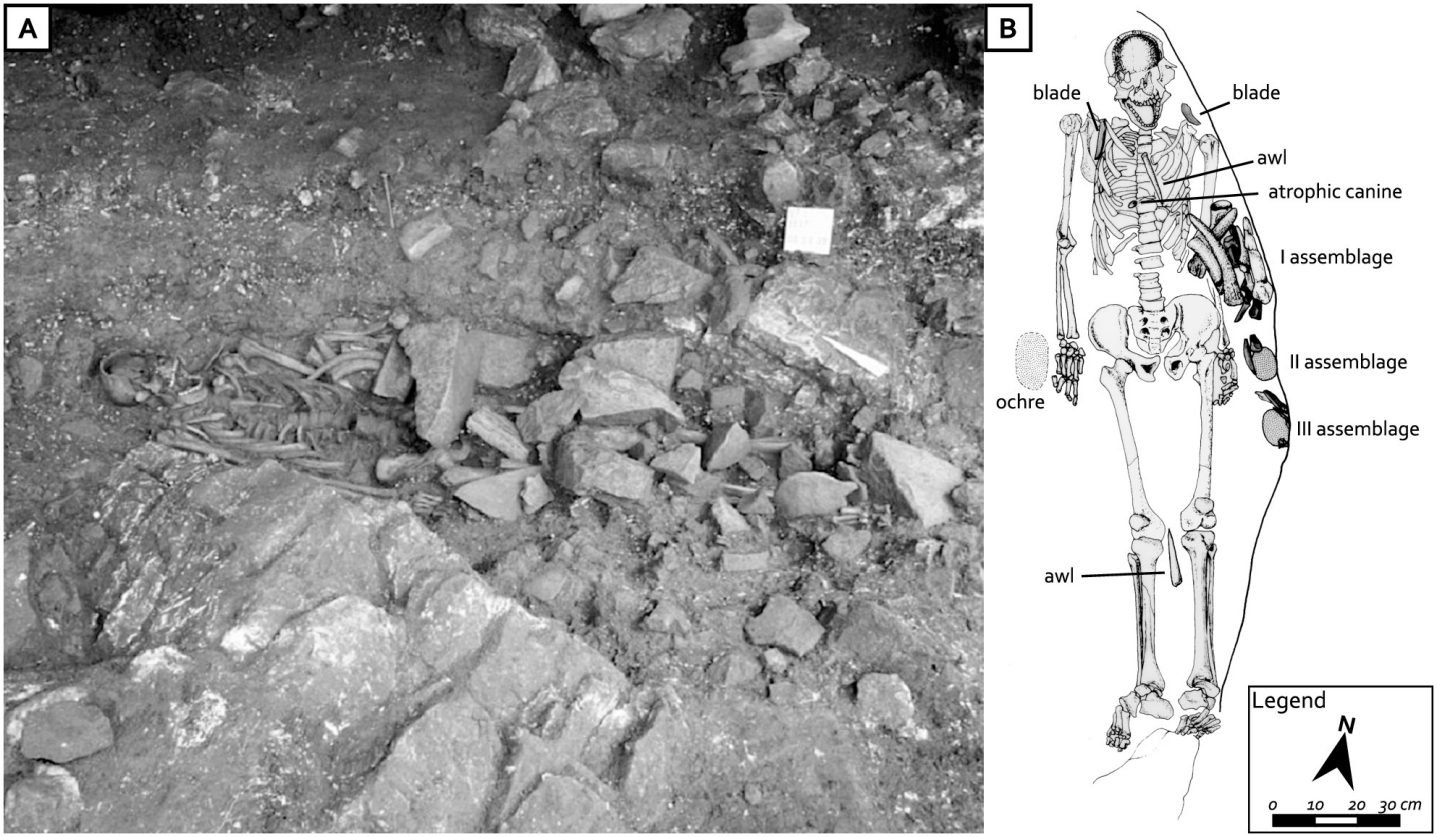
The burial of Mondeval de Sora covered with selected stones (photo: A. Guerreschi) (A) and plan of the burial with location of the grave goods (B).

The Castelnovian burial was identified in 1987 and was oriented north-south and parallel to the wall of the boulder. The skeleton had been placed in an elliptical-shaped pit and naturally delimited by two dolomite boulders, in a supine position with outstretched limbs [40,41]. The feet were located on a stone at the base of the pit, and the left hand was next to the body with the fingers slightly bent, as if the individual had been clutching an object at the time of the burial, perhaps a spear (or a bow?) that had subsequently decayed. The lower part of the body, from the pelvis downwards, had been covered with stones, apparently collected in the area surrounding the site (Fig 2A).

Paleoanthropological analyses carried out in past years determined that it was a human skeleton of a robust male individual around 40 years old and 167 cm tall and that he suffered from a polyostotic dysplasia (Rosy-Cajal disease), which belongs to a group of rare illness classified as pagetoid [33,42]. Studies on his dentition showed the presence of wear associated with the extra-masticatory use of teeth [33,42–44]. More recent work has focused on the biomechanics of the tibiofibular complex. Despite the degree of bone abnormality related to the disease, he suffered this research revealed a good degree of mobility, which is “compatible with the expected subsistence based on seasonal high-altitude hunting” [45]. The dietary habits of the individual have also been investigated through stable isotopes (δ13C; δ15N), which seem to indicate the possible integration of terrestrial and freshwater resources [46].

With regards to the age of the burial, one AMS radiocarbon date from the skeleton yielded a result of 7425±55 years B.P. (OxA-7468) (6430–6210 BCE). Of the four charcoal samples (collected within the sediment filling of the burial pit), one is chronologically close to this date (7330±50 B.P., R-1939, 6355–6065 BCE). Another is older (8380±70 B.P., R-1937, 7580–7195 BCE) and possibly indicates that the charcoal sample belonged to an older occupation layer, which would have been disturbed during the excavation of this burial pit. Finally, two dates are much more recent (5875±60 B.P., R-1941, 4900–4560 BCE and 4160±55 B.P., R-1936, 2890–2580 BCE). One suggestion is that these may come from samples of a more recent occupation phase, having seeped inside the burial pit. What is clear from this sequence of dates is that the area served as a primary place of activity for thousands of years [33,35–39].

We now turn to the burial itself. A small patch of red ochre was identified near the hand of the individual; in addition, 60 items had been carefully arranged near different areas of the body [41] (Fig 2B). The type of objects and their position with respect to the skeleton point to their role in a type of funerary ritual [24,41]. In particular, seven pierced red deer atrophic canines, collected in the upper part of the body, are considered to be the only ornamental objects accompanying the deceased, while a symbolic value has been attributed to the three blades placed above each shoulder and below the head respectively. Two awls, one found on the sternum and one between the knees, were likely to have been used for securing a (leather?) covering around the man. Lastly, three groups of various objects (grave assemblages I, II, and III) were documented along the left side of the skeleton, possibly indicating that they were originally located into three different bags made of organic material. The first grave assemblage (I), placed near the forearm, was composed of 34 objects, 22 of which are lithic flaked artifacts, 3 deeply altered limestone/dolomite pebbles, and 9 osseous artifacts. The second (II) and third (III) grave assemblages were found lower down, at the height of the left hand. The second (II) consisted of 3 elements: a lump of organic substances composed of propolis and a significant fraction of resin from pines (*Pinus sylvestris-mugo*) and spruce (*Picea* sp.), and two chert artifacts. The third (III) grave assemblages was made up of 11 items, including an agglomerate similar to the one found in the second group but with richer content of propolis, a boar tusk, and nine lithic artifacts. Due to the prevailing presence of resins, one interpretation is that the lump found in the second assemblage was used as glue, while the dominance of propolis in the other lump might indicate that it had a medicinal use (antibiotic, anesthetic or healing) [47].

### The lithic items from the burial furnishing

**Fig 3:**
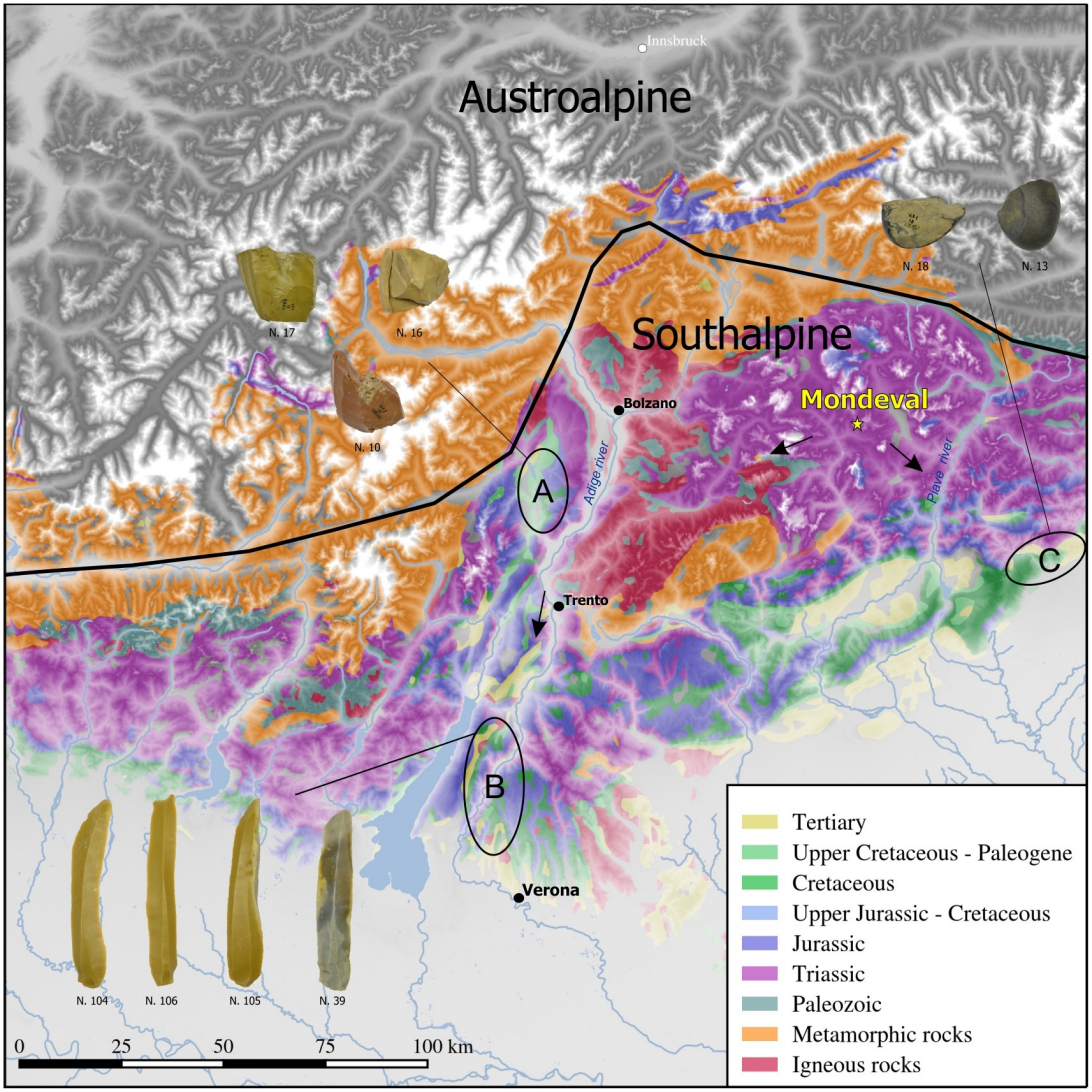
Provenance areas of the chert artifacts placed in the Mondeval burial. A = Non valley; B = Baldo-Lessini; C = Eastern Friulan foothills. Some representative artifacts from the grave good assemblage according to their provenance areas (for artefacts identification numbers see Table 1).

In their whole, 36 lithic artifacts formed the burial furnishing. As reported above, some of these were disposed in isolated locations (above the shoulders and under the head) while others were grouped into the three grave assemblages (I, II, and III) located along the left side of the body (Table 1). All of them were obtained from allochthonous chert sources, referable to the Jurassic and Cretaceous formations (Fonzaso, Maiolica, Scaglia Variegata Alpina and Scaglia Rossa) outcropping in the Venetian Prealps (Table 2) [48,49]. The greatest part of the exploited raw materials are high-quality cherts whose outcrops are located along and in the surroundings of the Adige valley, specifically in the Non valley and between the Baldo and Lessini chains, 75 to 150 km to the west/south-west of Mondeval de Sora [25]. The attribution of a few artifacts is more doubtful. Both the petrographic determinations and the morphological features of the three well-rounded cobbles and a few similar artifacts suggest acquisition from the Tertiary Flysch/Molasse deposits outcropping in the Eastern Friulian foothills between the Piave and the Tagliamento rivers thus updating previous determinations [25] (Fig 3).

**Table 1:** Mondeval de Sora, Castelnovian burial, composition of the lithic assemblage.

Position*	N.†	Length	Width	Thickn.	Raw material	Technology/typology	Refitting	Use wear traces
**Head**	-	105	17	5	SVA yellow	blade (abrupt retouch at the distal end)		hard animal (antler)/scraping
**Shoulder**	48	104	29	5	SVA yellow	blade		no use wear identified
**Shoulder**	49	106	15	7	SVA yellow	blade		no use wear identified
**I**	3	-56	18	5	MAI light grey	fragmented blade (prox. part) denticulated retouch (*cf*. Montbani)		hafting
**I**	7	62	45	33	SVA green	pre-core	n. 22 (opening flake—partial crest)	-
**I**	10	38	62	32	SR red	bladelet core		-
**I**	11	27	13	4,5	SR brown bicolor	end-scraper on a bladelet		no use wear identified
**I**	13	44	40	27	FON dark grey	pre-core		-
**I**	14	-74	17	5	SVA yellow	fragmented blade (prox. part) denticulated retouch (*cf*. Montbani)		hard material/scraping
**I**	15	36	23	21	SVA green	bladelet core		-
**I**	16	38	41	22	SR brown bicolorA	bladelet core	n. 23 (flake), n. 28–212697 (flake), n. 57 (néo-crête)	-
**I**	17	54	61	31	SVA green	bladelet core		-
**I**	18	35	56	31	FON light grey	flake core		-
**I**	19	29	43	33	FON dark grey	bladelet core		-
**I**	20	-64	17	6	SVA yellow	fragmented blade (distal part), denticulated retouch (*cf*. Montbani)		hard animal (antler)
**I**	22	30	26	9	SVA green	opening flake (partial crest)	n. 7 (pre-core)	not examined
**I**	23	37	32	12,5	SR brown bicolor	maintenance flake	n. 16 (core), n.28 (flake), n. 57 (néo-crête)	no use wear identified
**I**	24	65	17	7	FON dark grey	partial *néo-crête* (lateral marginal retouch)	n. 43 (sous-crete)	hard material (soft green wood?)/scraping
**I**	26	-54	14	5	FON light grey	*sous-crête* blade (distal part)		no use wear identified
**I**	27	31	21	16	FON dark grey	bladelet core		-
**I**	28	32	31	10	SR brown bicolor	maintenance flake	n. 16 (core), n. 23 (flake), n. 57 (néo-crête)	no use wear identified
**I**	31	-37	13	3	SVA yellow	fragmented blade (distal part)		soft material/scraping
**I**	50	36	11	4	SVA yellow	lateral bladelet		no use wear identified
**I**	56	15	19	4	FON dark grey	flake		not examined
**I**	57	38	11	3,5	SR brown bicolor	partial *néo-crête* on a bladelet	n. 16 (core), n.28 (flake), n. 23 (maintenance flake)	no use wear identified
**II**	33	109	21	6	SR red	blade		no use wear identified
**II**	34	30	47	25	SR brown bicolor	retouched flake (trihedral shaped tool)		striking hard material?
**III**	36	58	40	20	SR red	scraper on a flake		Polished area—leather processing?
**III**	37	71	16	6	MAI light grey	blade-burin on a truncation opposed to a truncation		undet. material/cut-engrave + semi-dry skin/ cutting
**III**	38	-38	6	2	SVA yellow	lateral blade (mesial part)		no use wear identified
**III**	39	92	15	6	MAI light grey	blade		soft animal/longitudinal
**III**	40	90	18	5	SVA yellow	blade		soft plant/transversal
**III**	41	-76	16	4	SVA green	fragmented blade (proximal part)		soft material/cutting
**III**	42	?	?	?	SVA yellow	fragmented bladelet		not examined
**III**	43	-33	22	3	FON darl grey	*sous-crête* (fragment)	n. 24 (partial neo-crest)	no use wear identified
**III?**	55	-33	15	9	undetermined	undetermined		no use wear identified

* I = first burial assemblage; II = second burial assemblage; III = third burial assemblage;

^†^ number assigned to each item at the time of discovery;

^‡^ geological formations: FON = Fonzaso; MAI = Maiolica, SVA = Scaglia Variegata Alpina, SR = Scaglia Rossa,.

**Table 2:** Mondeval de Sora, Castelnovian burial, petrographic features of the lithic items.

Formation	Age	Provenance area	Cortex	Color Munsell	Micropaleo	Microfacies
Fonzaso dark grey	MJ	Upper Friulan plain	cobbles	N/4; N/5	rad, ssp, pbiv, cri, bfor, bcl	Wack and pack with rad, ssp and pbiv
Fonzaso light grey	MJ	Eastern Friulian foothills	cobbles	2.5GY 7/1	rad, ssp, pbiv, cri, bfor, bcl	Wack and pack with rad, ssp and pbiv
Maiolica light grey	UJ-LC	Baldo-Lessini	undet.	10YR 5/1	rad, clp, ssp	wack and pack with dominant rad
Scaglia Variegata Alpina yellow	MC	Baldo-Lessini	outcrops	2.5Y 5/4	rad, pfor, ssp	wack and pack with rad and pfor
Scaglia Variegata Alpina green	MC	Non valley	outcrops	2.5Y 4/4	rad, pfor, ssp	wack and pack with rad and pfor
Scaglia Rossa brown	UC	Non valley	outcrops	10YR 5/4, 10R 4/2	rad, pfor, ssp	wack and pack with rad and pfor
Scaglia Rossa red	UC	Non valley	outcrops	10R 4/3	rad, pfor, ssp	wack and pack with rad and pfor

MJ: Middle Jurassic; UJ: Upper Jurassic; LC: Lower Cretaceous; MC: Middle Cretaceous; UC: Upper Cretaceous; rad: radiolarians, ssp: sponge spicules; cal: calpionellids; cri: crinoids; pbiv: pelagic bivalves; bfor: benthic foraminifera; pfor: planktic foraminifera; bcl: bioclasts; wack: wackestone; pack: packstone; grain: grainstone.

**Fig 4:**
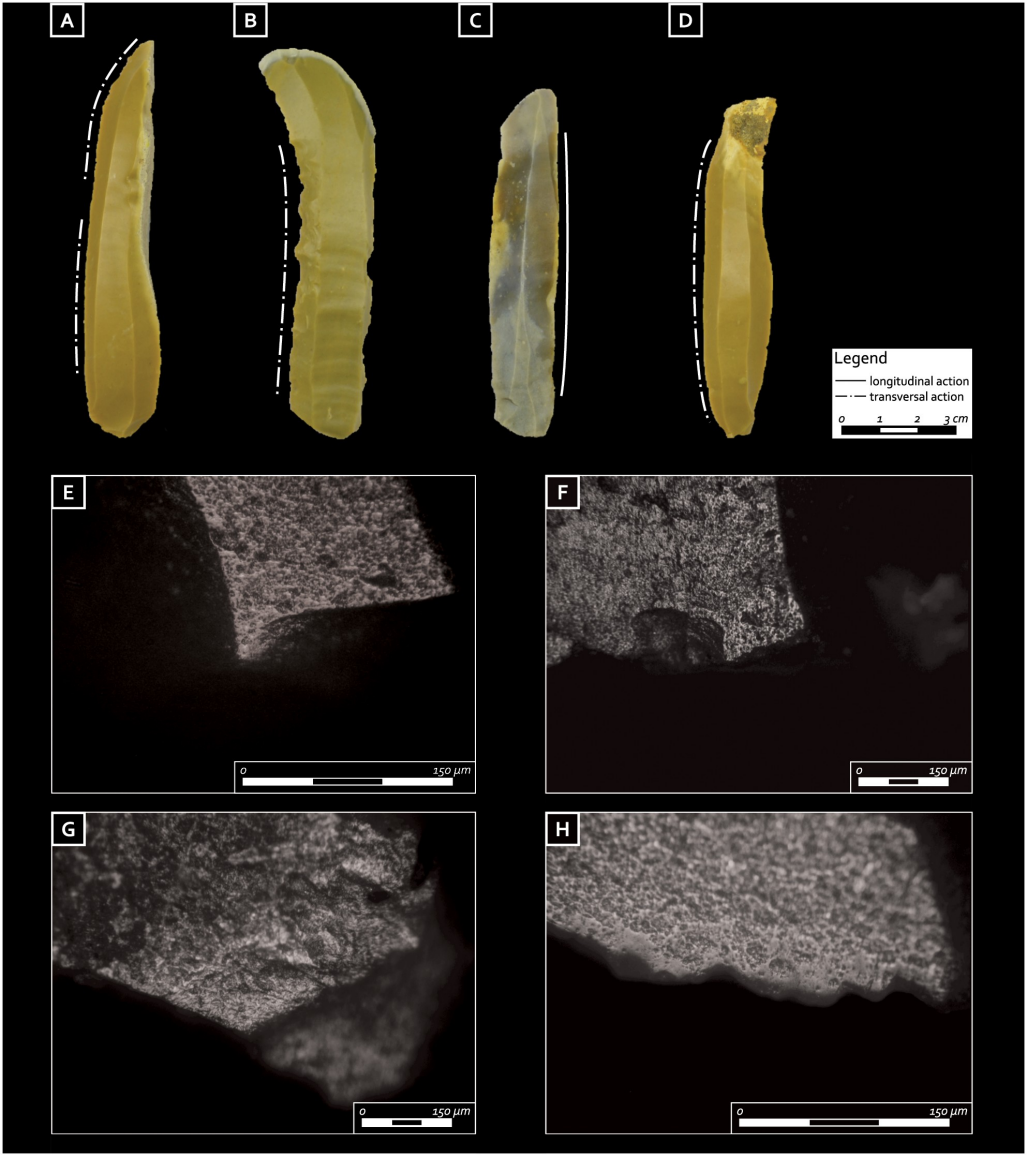
Blades from the burial furnishing: A, E) Blade located under the head, yellow Scaglia Variegata Alpina-(Baldo-Lessini) and micro-polish from scraping hard animal materials (antler) (magnification 200X); B, F) first burial assemblage: notched blade, yellow Scaglia Variegata Alpina (Baldo-Lessini) and micro-polish from scraping hard animal materials (antler?) (magnification 100X); C, G) third burial assemblage: blade, light grey Maiolica (Baldo-Lessini) and micro-polish from longitudinal motion on a soft material (magnification 100X); D, H) third burial assemblage: blade, yellow Scaglia Variegata Alpina, (Baldo-Lessini) and micro-polish from scraping soft plants (magnification 200X) (photos D. Visentin and S. Ziggiotti).

The two standardized chert blades placed above the shoulders and the one under the head (Table 1, Fig 3, no. 104, 105, 106) of the individual denote the knapper’s excellent technical skills. The items reach unusual dimensions being approximately 100 mm in length and 20 mm in width. Such dimensions are rarely if hardly ever found in habitation contexts, as shown for example, by the typometric values of the lithic assemblage from the Castelnovian levels of Romagnano Loc III, which never overpass 60 mm in length [34]. These blades are also made from the same allochthonous chert (Scaglia Variegata Alpina) and possibly even from the same core (although attempts to refit them have proved unsuccessful). The morpho-technical features observed on the blades—i.e., their curved profile, thick and faceted butts, prominent bulbs, width between 18 and 20 mm, and thickness between 5 and 7 mm—indicate that they were extracted from the core using the punch technique (knapping by indirect percussion). Of the three blades only the one located under the head (Fig 4A) shows modifications due to intensive use along its left lateral side, notably semi-circular and step ending edge removals, together with a smooth and matt micro-polish with irregular limits and distribution (“fingering” distribution as defined by [50], p. 35) and transverse directionality, characteristic of scraping antler (Fig 4E)) (cf [49, 50] for an experimental comparison to the use wear and hafting traces identified on the archaeological chert tools mentioned in the paper). In contrast, the two blades located above the shoulders present well preserved, pristine and unused edges.

**Fig 5:**
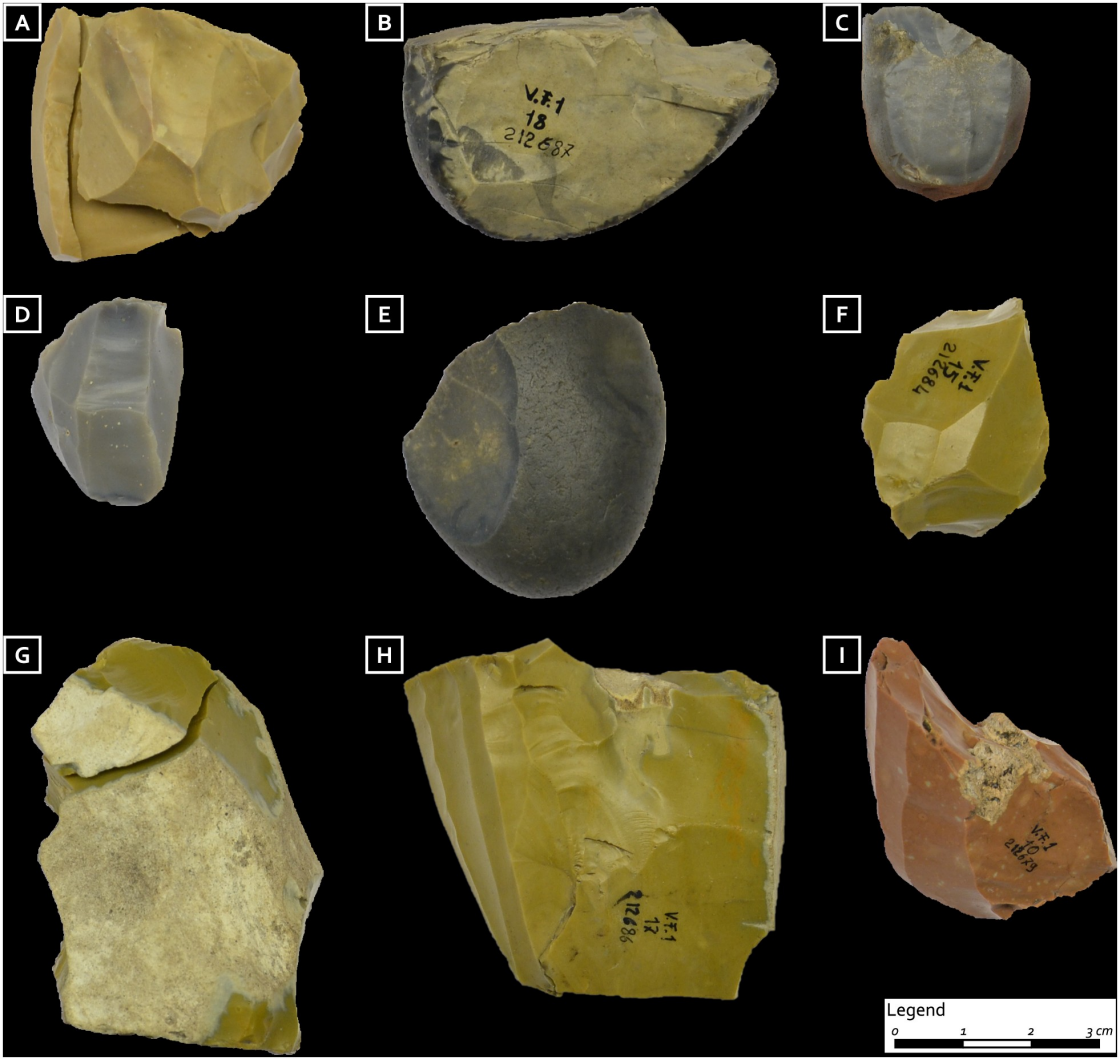
Cores from the first burial assemblage: A) bladelet core, brown bicolor Scaglia Rossa (Non Valley) with one refitted neo-crested bladelet and two maintenance flakes; B) flake core, dark grey Fonzaso, Eastern Friulian foothills; C-D) bladelet cores, dark grey Fonzaso, Eastern Friulian foothills; E) pre-core, dark grey Fonzaso, Eastern Friulian foothills (natural size); F) exhausted bladelet core, green Scaglia Variegata Alpina (Non valley); G) pre-core, green Scaglia Variegata Alpina (Non valley) with refitted opening flake; H) bladelet core, green Scaglia Variegata Alpina (Non valley); I) bladelet core, red Scaglia Rossa (Non valley) (photo: D. Visentin) (from Fontana et al. 2016b modified).

**Fig 6:**
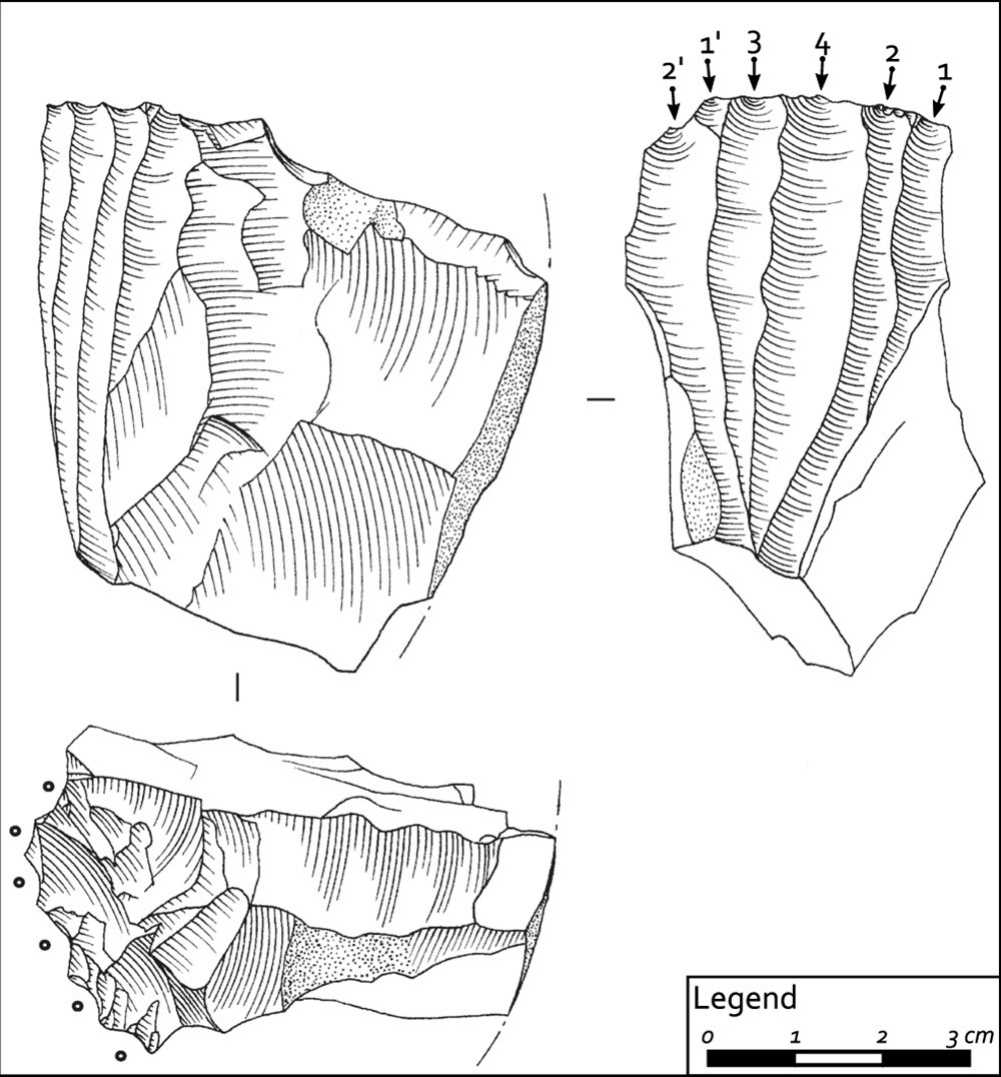
Bladelet core from I burial assemblage, green Scaglia Variegata Alpina (Non valley), showing evidence of pressure technique (drawing: F. Briois).

The lithic artifacts from the first grave assemblage (I) located on the left side of the body are represented by nine cores, six full debitage blades, two bladelets (a partial neo-crest and a backed bladelet), a small endscraper, and four flakes (which include one opening and two maintenance elements) (Table 1). Among the nine cores (two of which are pre-cores, six are bladelet cores, and one is a flake core), five may be sourced to allochthonous cherts (green Scaglia Variegata Alpina and red and brown Scaglia Rossa of the Non valley) (Fig 3, no. 10, 16, 17 and Fig 5A and 5F–5I). The other four were produced from good quality chert (Fonzaso formation) available under the shape of rounded cobbles in the Flysch/Molasse deposits distributed along the eastern Friulian foothills (Fig 3, no. 13 and 18 and Fig 5B–5E). The use of pressure technique is strongly suggested for at least two of the bladelet cores, which have a faceted striking platform, an angle between the striking platform and the surface of debitage equal to or greater than 90° and nested negative bulbs corresponding to precise and well controlled removals (Figs 5A and 5H and 6). All of the six blades, only one of which is entire, have diagnostic characteristics of extraction by the punch technique, yet regardless of this similar manufacture, they are made on different chert sources (Table 1). Three show discontinuous irregular denticulated retouch (notched blades, cf. Montbani). Among them, one displays a slightly rounded edge opposite to the retouch and was probably used for cutting/scraping medium-hard materials; on the same denticulated blade, a pitted and grooved bright spot is visible on the dorsal proximal ridge which might be related to a hafting system [51]. The other two show evidence of scraping a more resistant material (unilateral, symmetric, medium size, feathered edge scarring); one of them also displays a micro-polish characterized by medium to high level of linkage, great contrast, smooth texture, bright aspect, domed/flat topography, somehow pitted, linear perpendicular directionality and irregular limit and distribution. These features are consistent with antler scraping (Fig 4B and 4F).

Another blade (a partial neo-crest) is characterized by a marginal retouch and an active zone with slightly rounded edge, irregular but continuous symmetric feathered edge removals, associated with micro-polish, on protruding points of the ventral face, with medium/great contrast, smooth texture, bright aspects, domed topography, marginal width; these traces have been interpreted as evidence of scraping hard materials, possibly wood. The last blade displaying traces of use presents edge scarring on its left side (unilateral, symmetric, feathered scars) together with a slight rounding of the edge and a generic weak polish, suggesting that it was employed for scraping soft material. No traces of wear were identified on the sixth blade, the bladelets, and the small frontal endscraper. One of the two bladelets (a neo-crest made on Scaglia Rossa bicolor) has been refitted on one of the small lamellar cores together with a maintenance flake and a flake (Fig 5A; Table I), while an opening flake (green Scaglia Variegata Alpina) has been refitted on one of the two pre-cores (Fig 5G; Table I).

In sum, the artifacts from this I assemblage attest to both the use of pressure technique (for bladelet extraction) and of the punch technique (for blade extraction) corresponding to two separate *chaînes opératoires*. Five of the blades display use wear traces, all related to transversal motions, i.e. scraping mostly of hard materials.

**Fig 7:**
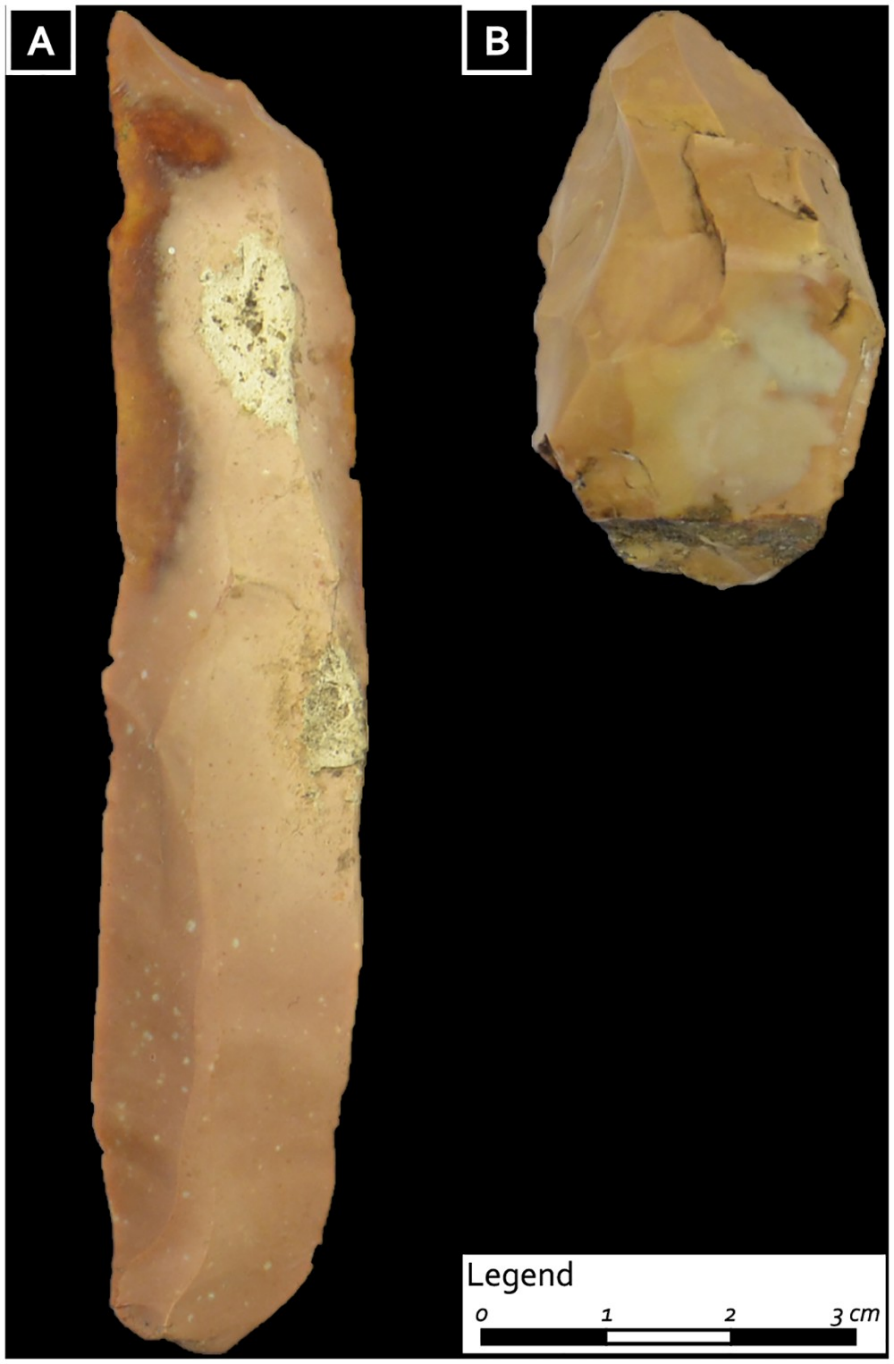
Lithic tools from II burial assemblage: A) blade, red Scaglia Rossa (Non valley); B) trihedral shaped tool, SR brown bicolor (Non valley) (natural size) (photo: D. Visentin) (from Fontana et al. 2016b).

The second grave assemblage (II) is represented by two items, both made with the Scaglia Rossa from the Non valley and with surfaces partially altered by contact with the organic lump accompanying them. One is a lateral blade extracted by the punch technique, characterized by a large width and thickness, a faceted butt and a pronounced percussion bulb, and with no evidence of use (Fig 7A). The second one is a thick flake with a trihedral shape and a bifacial coarse retouch at one of the extremities, creating a rounded “nose” on which edge removals are superposed; this suggests that it was used for striking hard materials (such as a strike-a-light or a retoucher) (Fig 7B). Tools with a similar morphology and wear were found at the Castelnovian sites of Laghetti del Crestoso and Fienile Rossino (south-central Alpine area) [52].

All the lithic items that comprise the third grave assemblage (III) are variably covered and altered by contact with the lump of organic substances accompanying them (Table 1). Four blades (two of which are whole), two broken bladelets, a small flake, a modified blade (typologically a “burin on a truncation”), and a retouched flake (typologically a “scraper”) make-up this group. The four blades are made with allochthonous cherts and all their features indicate extraction by the punch technique.

One of them presents a functional area along the left edge, displaying small, bilateral, half-moon shaped and feathered edge removals, slightly rounding and a micro-polish with little contrast, rough and greasy texture, dull brightness and corrugated topography, attributed to a longitudinal motion on a soft animal material (Fig 4C and 4G). On another one a micro- polish was spotted on both dorsal and ventral surfaces, more developed on the ventral one, bright, with smooth and matt texture, medium contrast, domed topography, from marginal to more invasive width, with linear perpendicular directionality and perpendicular striations; these traces are consistent with transversal motion on a soft plant [49,50, 66,73] (Fig 4D and 4H). The burin presents a functional area on the distal truncated end (appearing very rounded), which can be associated with cutting/engraving an undetermined material while its right edge, slightly rounded, displays small elongated edge removals with a generic weak polish on dorsal and ventral surfaces, rough and matt texture, dull brightness, corrugated topography and linear parallel directionality; such evidence can be attributed to cutting semi-dry skin. The scraper is characterized by a very rounded area on its distal margin, suggesting that it was involved in leather processing. All artifacts with traces of wear from this assemblage indicate the processing of soft materials [49,50].

### The osseous items from the burial furnishing

**Fig 8:**
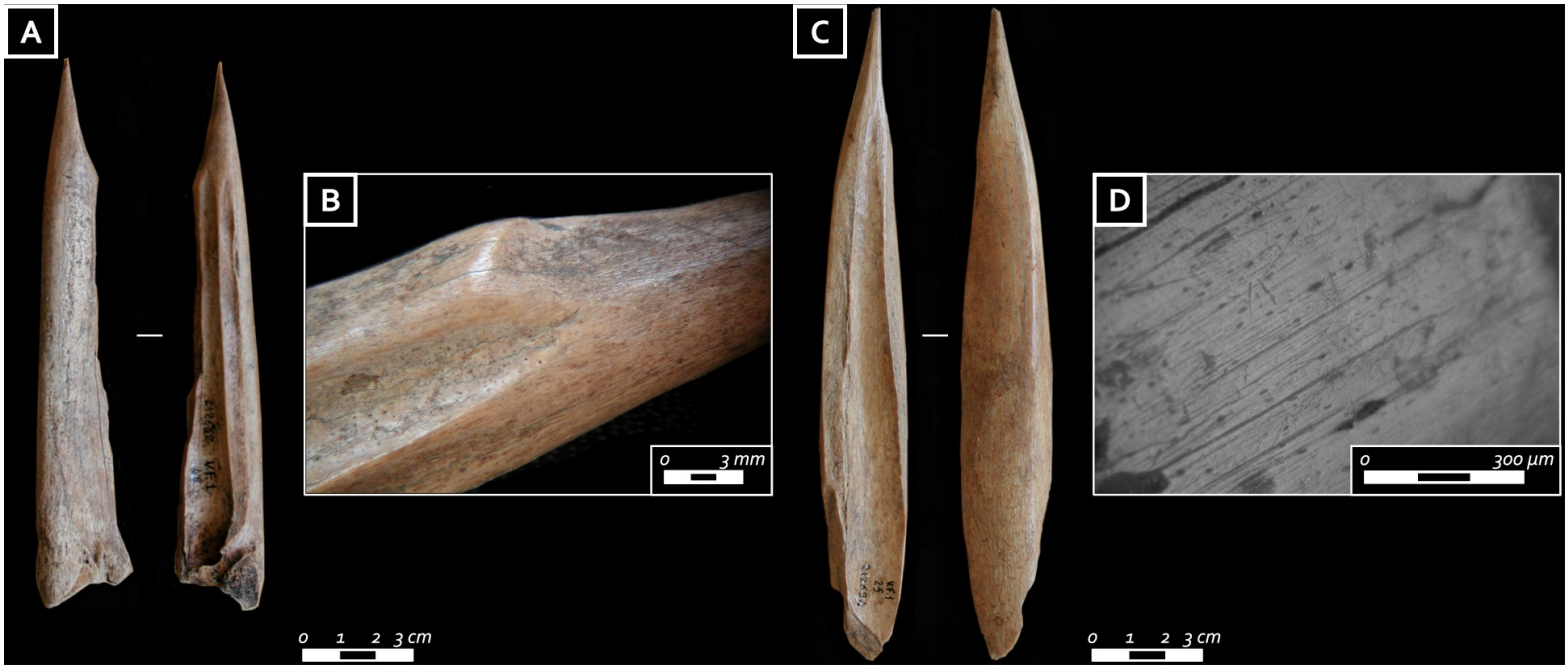
A) Awl from elk vestigial metapodial from the first grave goods assemblage; B) rounding on the distal part of the awl on elk metapodial); C) awl from red deer metatarsal from the first grave goods assemblage; D) use wear traces on the distal part of the awl from red deer metatarsal. Note the developed rounding with flat profile, small long depressions and longitudinal striations (photo: E. Cristiani).

Osseous artifacts in the burial furnishing amount to19 items (Table 3). Some appear isolated (the seven red deer atrophic canines and the two awls respectively located between the knees and on the sternum) while others are part of two of the three grave assemblages (I and III) located on the left side of the body. The awl found on the sternum of the buried individual was obtained from a vestigial elk metapodial (Fig 8A) and the one between the knees from a red deer metatarsal (Fig 8C). The elk awl shows regular wear, likely from flint scraping; in contrast, the impact cones on the ventral surface of the red deer awl reveal that it was extracted from the diaphysis through indirect percussion and later finished with a stone tool. Use wear and prehension traces are well developed on both artifacts. In particular, a developed rounding with a flat profile, the presence of small long depressions, and longitudinal striations on the mesial and distal part of the awls suggest that both tools were used in longitudinal motion on soft plants [53] (Fig 8B and 8D).

**Table 3:** Mondeval de Sora, Castelnovian burial, composition of the osseous assemblage.

Position	Raw material	Description	Functional interpretation
**Sternum**	Elk vestigial metapodial	Awl	Longitudinal motion on soft plants
**Knees**	Red deer metatarsal	Awl	Longitudinal motion on soft plants
**Upper part of the body**	Red deer atrophic canines	Perforated ornaments (7 items)	Pendants
**I**	Antler tine	Punch/pressor	Flint knapping in indirect percussion/pressure
**I**	Antler tine	Punch/pressor	Flint knapping in indirect percussion/pressure
**I**	Antler tine	Punch/pressor	Flint knapping in indirect percussion/pressure
**I**	Antler tine	Punch?	No functional traces
**I**	Red deer antler compact tissue	Harpoon with alternate barbs, basal bilateral gorge and a beveled base	Detachable head harpoon with traces of prolonged use and insertion in a shaft
**I**	Antler tip	Awl	Perforation of hard material
**I**	Long bone diaphysis	Awl	Longitudinal motion on soft plants
**I**	Red deer scapula	Multipurpose tool with bevel as well as diffuse active parts	Digging/spading activities as well as a presser
**I**	Red deer thoracic vertebra	Tool with diffuse active part	Shaft straightener
**III**	Wild boar tusk	Burin	No functional traces

**Fig 9:**
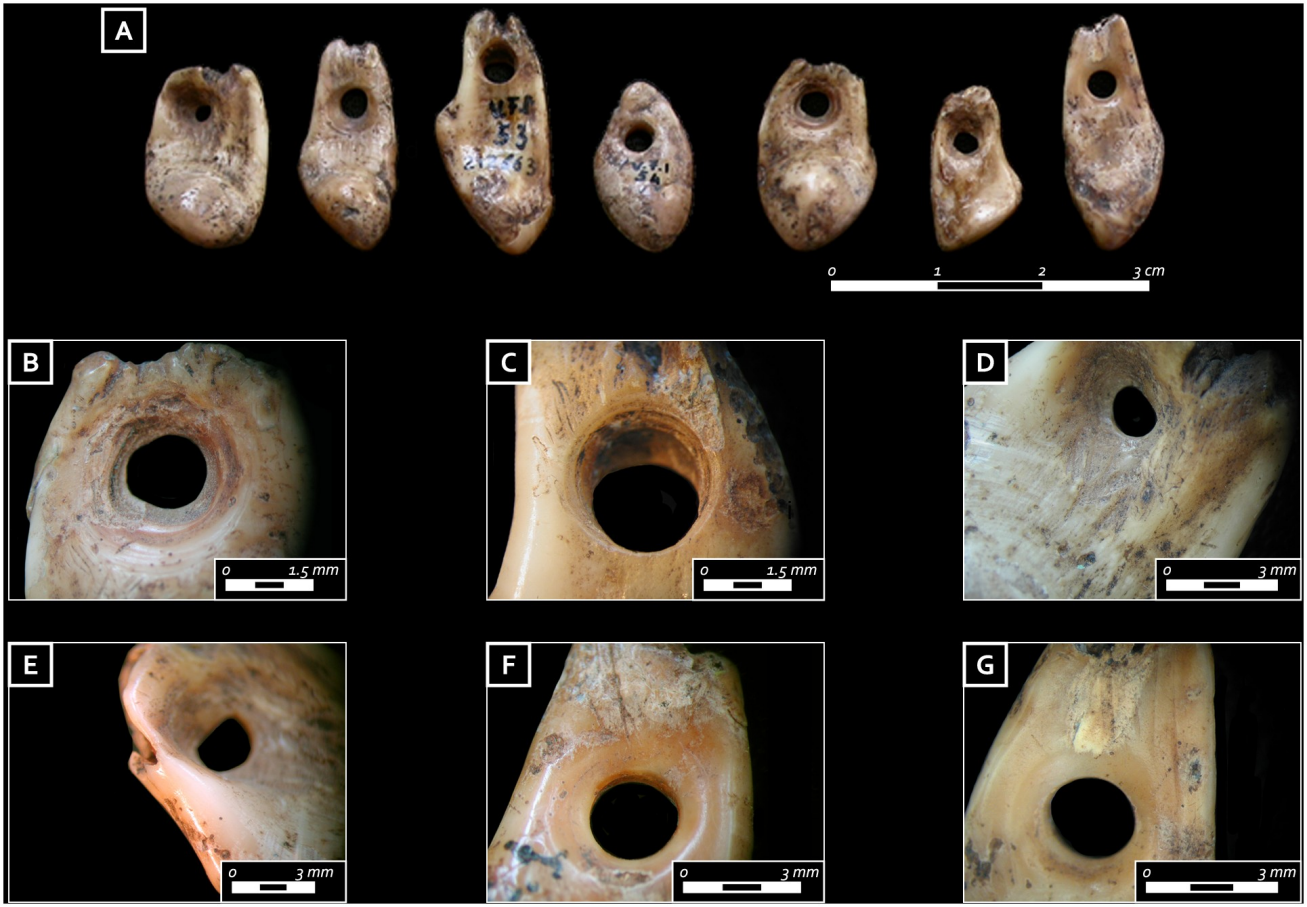
A) Ensemble of ornamental perforated red deer teeth; B) close-up on the hole of an ornamental tooth characterized by a biconical section and an irregular profile); C) symmetrical hole of another ornamental tooth characterized by a cylindrical section probably produced using a mechanical drill; D) close-up on one ornamental tooth with thinning traces produced on the root before drilling; E) rounding on the lateral sides of the perforation of an ornamental tooth; F) rounding on the lateral sides of the perforation of another ornamental tooth. Note how the developed rounding has deleted the previous thinning and drilling traces; G) close up of the other side of the previous tooth ornament showing the development of the rounding traces. Note how the use traces have worn out technological marks (photo: E. Cristiani).

The seven perforated red deer atrophic canines found on the upper part of the skeleton appear to be from seven different (i.e., individual) males (Fig 9A; for identification criteria, see [54]. Their roots were slightly thinned and/or grooved using a flint tool and then perforated (Fig 9B and 9D). The holes are mostly characterized by a biconical section and an irregular profile. On the basis of experimental comparison, all these features can be associated with a free-hand rotation, produced using a pointed flint tool on both sides of the teeth. Only two canines show symmetrical holes with cylindrical sections (Fig 9C) [55]. Similar perforations have experimentally been obtained using a mechanical drill (e.g., bow or a pump drill) mounted with a retouched pointed tool, such as a backed point and/or a perforator. Both types of tools were found in the Late Mesolithic layers of the rock-shelter sites of the Adige valley, such as Riparo Gaban [31], Riparo Pradestel [56] and Riparo Romagnano Loc III [57] while perforators are rarely documented at upland sites [16,52]. Use wear traces such as faceting and rounding are predominately found along one side of the respective root, inside the hole and around the crown. This pattern indicates that these ornaments would have been worn as pendants (Fig 9E–9G), thus possibly being part of a necklace as also indicated by their position in the burial pit. Yet, one canine shows use wear traces on both sides of the hole and the root, and thus potentially was attached or worn differently. The varying degree of use wear characterizing the perforated deer canines indicates a range of life histories.

**Fig 10:**
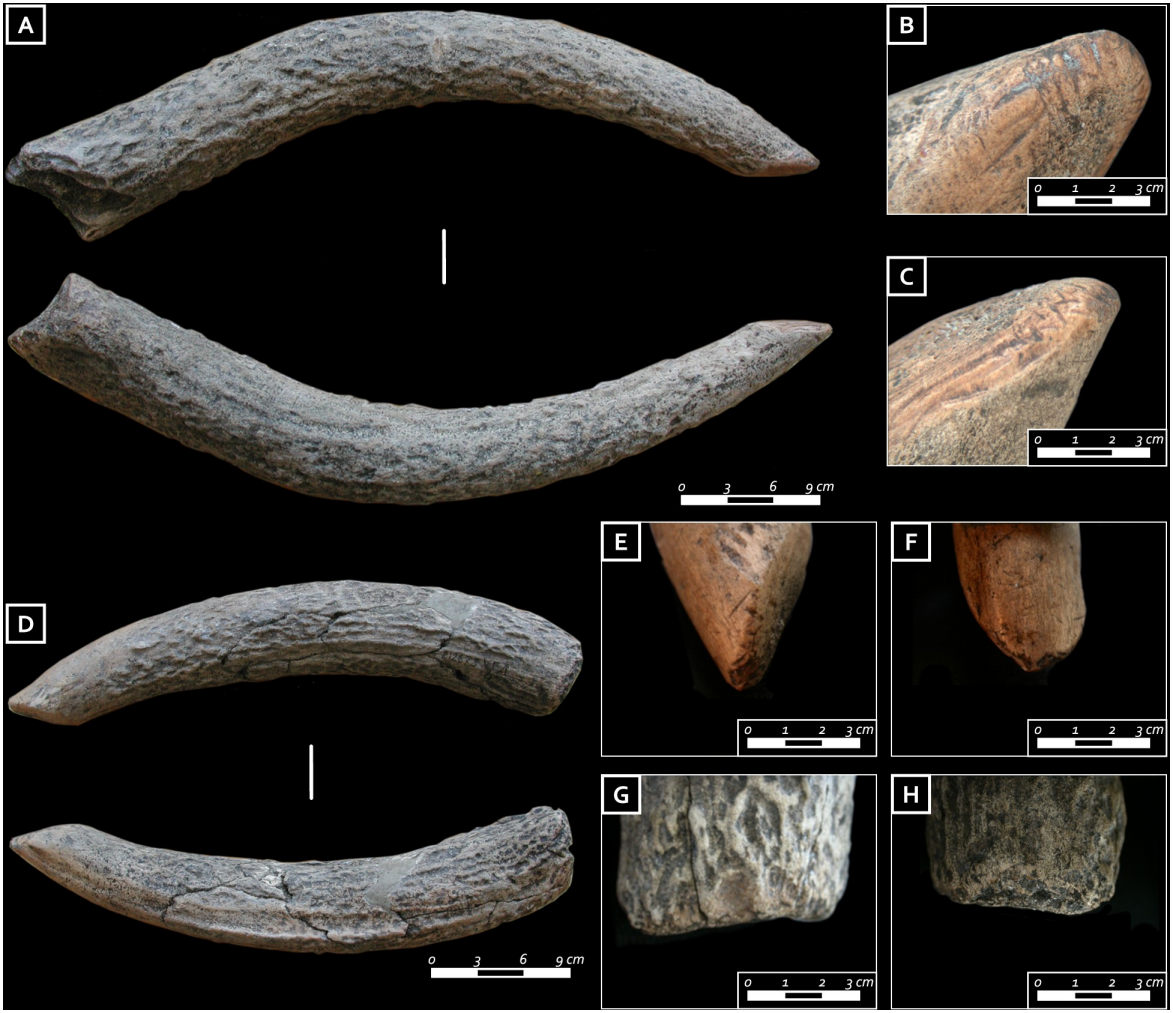
A) Deliberately worked antler tine from the first grave good assemblage; B, C) close-up of the striations and compression marks characterizing the tip of the antler punch; D) antler tine from the first grave good assemblage; E, F) close-up of the striations and macro-retouch characterizing the tip of the antler punch; G, H) detail of the proximal part of the antler tine characterized by compression marks and use-retouches (photo: E. Cristiani).

The first grave assemblage (I) yielded nine bone and antler items, namely four antler tines, a bilateral harpoon, two awls, a red deer scapula, and a red deer thoracic vertebra. The four antler tines were extracted from the main beam by nicking followed by flexion, and their tips intentionally shaped. Three out of four tines show functional modifications. In particular, traces of rounding and flattening are visible all around the proximal ends (Fig 10H) while, in one case, rectangular stepped use-scars are identified (Fig 10G). Similar traces were documented on the butts of experimental punches used in indirect percussion with a tender (wooden) hammer tool as well as on antler tines used with pressure-flaking techniques [52]. The distal (active) tip ends are deformed and have flattened outlines, bearing functional traces in the form of wide and deep striations (Fig 9). These modifications are consistent with marks left by flint knapping. The association of the modifications located on the tips and proximal parts of these antler tools would be consistent with such an interpretation [58].

**Fig 11:**
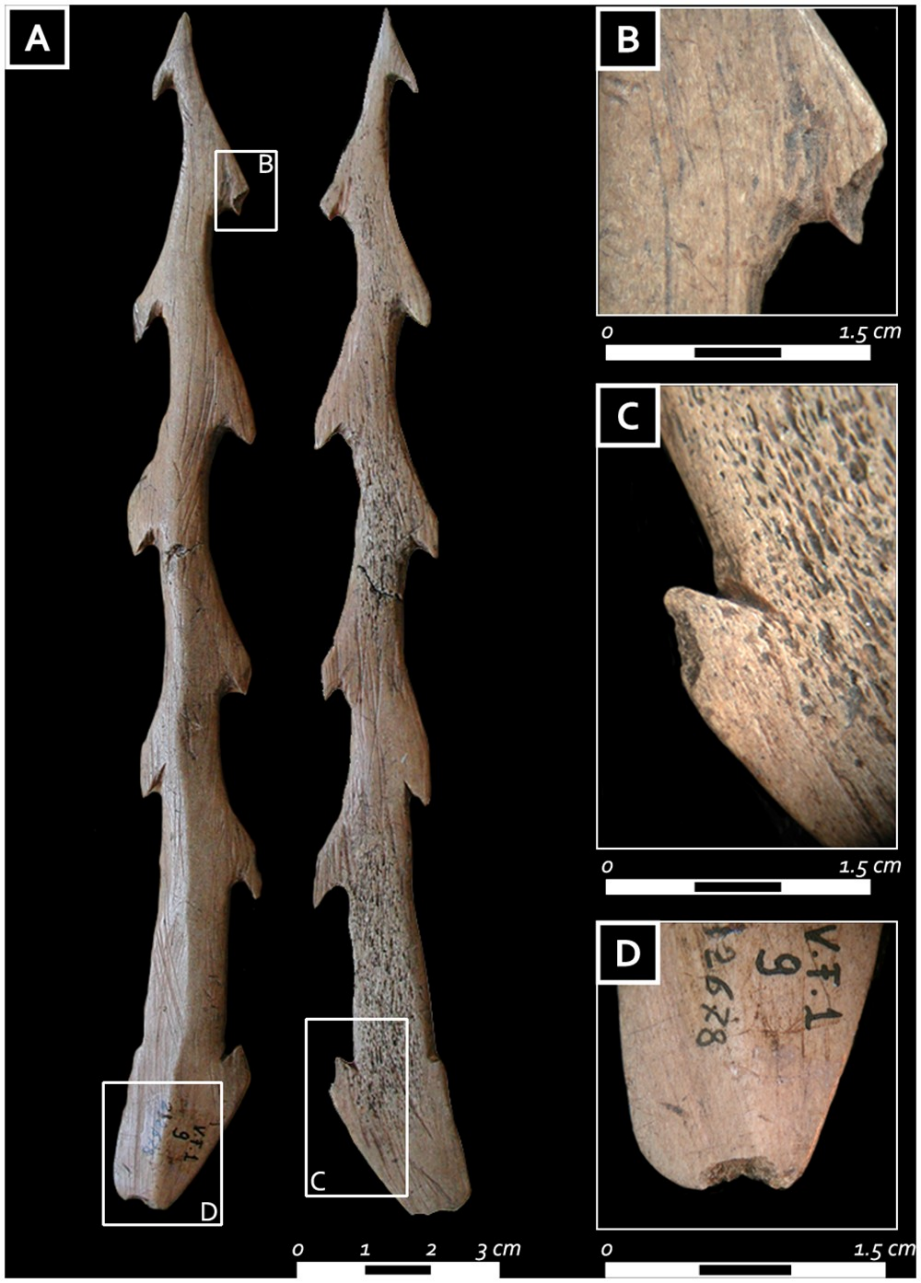
A) Harpoon from the first grave good assemblage; B) bending fracture and rounding on a lateral barb; C) bending fracture and rounding on one side of the gorge; D) compression marks and macro-scars along the base (photo: E. Cristiani).

The bilateral harpoon, made from the compact tissue of a red deer antler beam, is characterized by alternate convex barbs, basal bilateral gorge, and a beveled base (Fig 11A) [55]. The tool is whole, 187 mm long, and has a triangular cross-section. Although the final regularization of the surfaces through flint scraping would have erased traces of manufacturing related to the blank extraction, it is possible that the blank was removed from the compact tissue of the antler by longitudinal grooving, a technique already documented for the production of morphologically comparable late Mesolithic harpoons in the Alpine region (e.g., Romagnano, Pradestel, Dos de la Forca; see [59]). Bilateral barbs and proximal gorges were preliminarily outlined and subsequently incised on their lateral side; the depressions were enhanced through chiseling. The latter technique also produced the harpoon’s sinuous outline. Use wear traces indicate that the tool was used before being placed in the burial. Surfaces are well rounded and two lateral barbs (one distal and one on the side of the gorge) show a bending fracture as well as rounding (Fig 11B–11C). Compression marks and macro-scars along the base (Fig 11D) indicate that it was probably fixed to the shaft with a line (similarly to detachable head harpoons in the ethnographic record; see [60–64].

One of the two awls was produced from an antler tip. The blank was extracted from the antler through nicking and snapping and, successively, finished by flint scraping. Clear functional modifications were identified on the distal end. In particular, five longitudinal grooves along the tip and a circumferential striated deformation produced by rotation have been documented. These use wear patterns find parallels in experimental archaeology, notably for hard material processing (e.g., wood) (cf [65] for an experimental comparison about use wear traces on osseous tools). The proximal part of the tool shows traces of prolonged prehension. Similar to the specimen found between the knees (see above), the second awl was produced by indirect percussion, standardized by flint scraping and was subsequently used in plant-processing activities (cf [66] for diagnostic criteria for interpreting Mesolithic osseous technology; cf. [67] and [68] for experimental comparison about plant-related use wear traces on bone surfaces).

**Fig 12:**
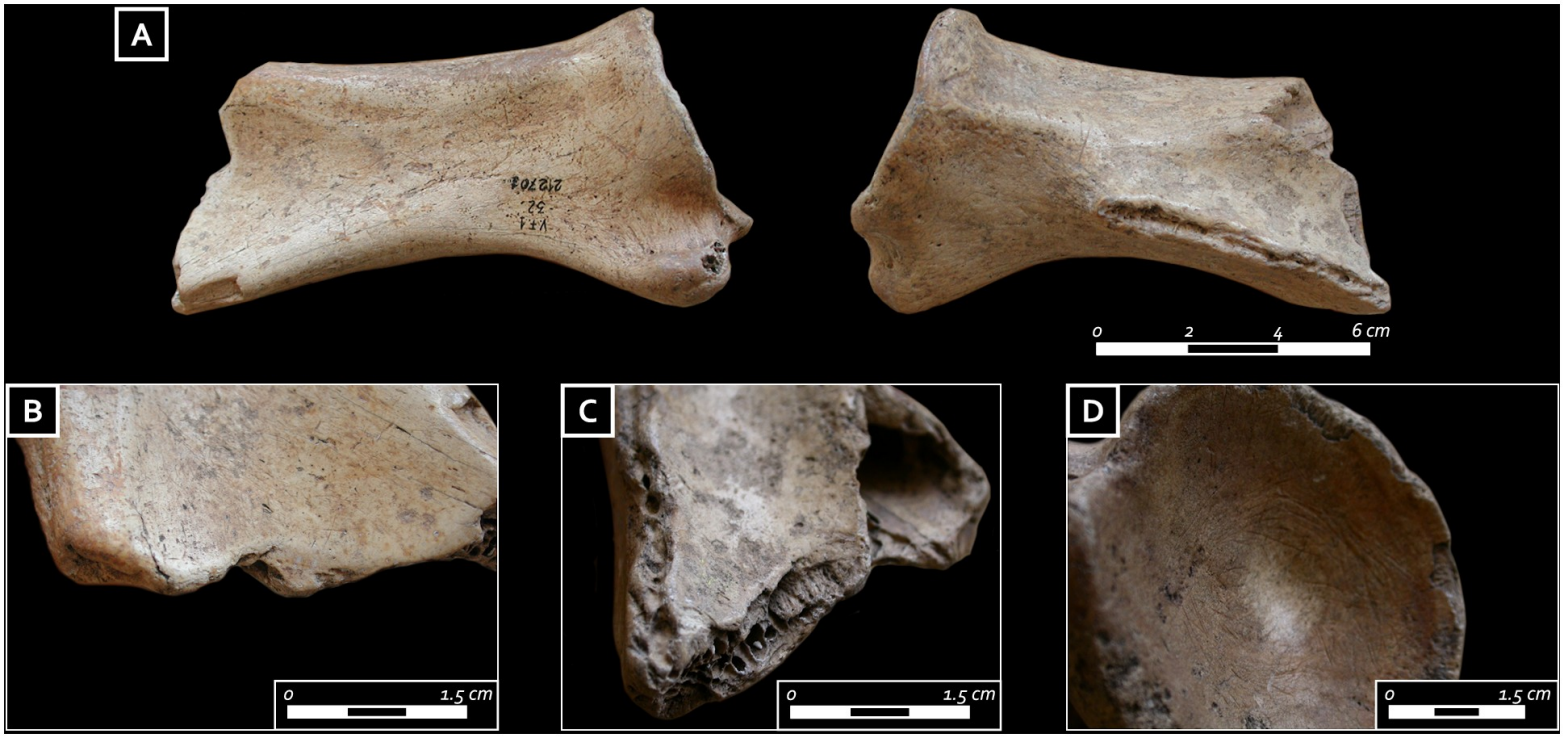
A) Red deer scapula from the first grave good assemblage; B) close-up of the rounding and faceting located on the distal edge; C) close-up of the use-retouches and faceting located on the distal edge; D) Detail of circular compression marks at the center of the glenoid cavity and non-continuous concentric striations (photo: E. Cristiani).

The spine of the red deer scapula was removed through percussion and then rounded as a result of the subsequent intensive use (Fig 12A). In addition, there is clear evidence of functional alterations of the distal edge, such as use-retouches, modification of the outline, deep transversal striations, rounding, and faceting (Fig 12B–12C). Rounding is also located in areas corresponding to the glenoid cavity and on the coracoid process. The traces are well developed and we suggest that the tool has a long life history and was probably used in digging or spading activities. However, the scapula probably represented a multipurpose tool as circular compression marks (Fig 12D) and non-continuous concentric striations at the center of the glenoid cavity hint at other uses (for example, use as a presser of a bow-drill was already suggested by previous studies [69]). Finally, the thoracic vertebra shows developed compression traces in the area between the two caudal articular facets, for which it was suggested a utilization as a shaft straightener [69].

Lastly, the third grave assemblage (III) included a burin on a wild boar (*Sus scrofa*) tusk. The occlusal surface of the tooth was regularized through flint scraping in order to create a dihedral edge. No functional diagnostic traces were identified on it.

## Discussion

### About lifestyle

The analysis of raw materials, manufacturing techniques, and use wear traces of the lithic and osseous items that compose the burial goods of Mondeval de Sora offers some keys to help reconstructing the lifeways of Late Mesolithic groups settled in north-eastern Italy during the time-span included in the second half of the 7th millennium BCE.

As far as lithic implements are concerned, our analysis documents the use of cherts from diverse directions, to the west (Non Valley), the south-west (Bado-Lessini) and the south-east (Eastern Friulian foothills) of Mondeval de Sora over a wide area spanning between some tens and over one hundred kilometers as the crow flies (Fig 3). Such provisioning strategy is very different from the one attested in the Early Mesolithic occupation layers of the same site where the origin of cherts points to the Piave valley. Namely, the exclusive presence of allochthonous cherts of high quality in this burial, also documented among cores, is unique for a Mesolithic context in Northern Italy and suggests that these raw materials were obtained either by direct procurement or by exchange. Given the distances covered in different directions, it is unlikely that these materials were acquired within an embedded system [70]. All the lithic elements reflect a lamino-lamellar reduction sequence (Table I). Lamellar debitage is mainly represented by cores, some of which must have produced a few dozen bladelets, almost absent in this funerary deposit. The bladelets from full debitage were probably used outside, most probably for the production of geometric microliths, which are also absent here. These cores, therefore, seem to play a significant role by indicating that the deceased may have been practiced the bladelet debitage activity. As to the blades, some of them—namely the one located under the head, the two above the shoulders, and the one belonging to the second (II) grave assemblage of objects—are exceptional for their dimensions (over 100 mm in length). This evidence suggests that curated items such as these were part of “limited” production, perhaps pointing to exclusivity. Moreover, our analysis indicates that all the blades from the burial furnishing were produced by the punch technique, while bladelet cores show evidence of the use of the pressure technique. To our knowledge, this is the oldest evidence, at the same site, of the contemporary use of these two techniques for manufacturing two different categories of elongated blanks. Among the grave goods, the presence of slightly curved antler tines with characteristic functional modifications support this interpretation (see below). Although studies aimed at identifying knapping techniques for the production of Neolithic lithic assemblages in Northern Italy are still limited, it seems that the technical choices we documented for the burial of Mondeval predate a behavior that became widespread during the later period [71–73]. Finally, our functional analysis highlighted that almost all the laminar blanks were used prior to their placement in the burial pit, exception made for the two placed above the shoulders of the deceased. Identified wear traces reflect two main specialized activities (Table 1). A group of them—mostly from the first (I) assemblage and modified by a lateral retouch with notches (notched blades)—was utilized for working hard/medium-hard materials with a transversal motion. Such an observation fits well with the results obtained on laterally notched blades/lets (the so-called “Montbani bladelets”) from different sites of the Late Mesolithic in the Iberian peninsula and Southern France, which attest to an almost exclusive use for scraping, with a negative rake angle, different raw materials including soft plants with high silica content, soft plants with an abrasive component, wood, bone, and antler [29,74]. The second set of items—mostly from the third (III) assemblage and with unmodified lateral edges—was used for processing softer substances (both animal and vegetal), mostly with a longitudinal action.

Moving to the osseous assemblage, red deer bone and antler are the main raw materials represented in the burial, although an awl made from an elk metapodial and a wild boar incisor are also documented (Table 2). Within this corpus, some awls appear particularly significant as they were used for plant-based crafts, possibly in association with some of the blades from the third assemblage. Developed use wear on their surfaces are consistent with their utilization in the production of ropes, strings, and baskets manufactured from plant species available at lower altitudes, although we cannot exclude that they were also involved in the production/maintenance of nets and traps, i.e., the array of technical solutions connected to the exploitation of lake and freshwater resources (see [68,75,76] for an updated discussion about siliceous plant-working use wear on various artifacts and their involvement in hunter-gatherer crafts). A specific association of bone items with the acquisition of freshwater resources comes from the analysis of the harpoon associated with the first (I) grave assemblage. Similar tools recovered at various Late Mesolithic sites in the eastern Alps, namely in the Adige valley (Romagnano, Pradestel, Gaban, and Dos de la Forca), the Dinaric Alps, Central Switzerland indicate innovation in aquatic resource hunting [77]. Alpine areas are rich in lakes and rivers, and fishing as well as trapping animals linked to wetlands, such as beavers, might have represented an important activity carried out by Castelnovian groups [78,79] for a discussion of the faunal remains from Mesolithic sites of the Adige valley). Moreover, the possible integration of freshwater resources in the diet of the individual is not excluded on the basis of stable isotope analysis [46] and from the recovery of pike’s vertebras, together with those of other freshwater fish species, used as ornaments during the Mesolithic in the Eastern Alpine region [80]. In Central Switzerland and Austria, the tight correlation between harpoons and fishing practices is confirmed during the Late Mesolithic at Abri of Liesbergmühle VI [81], Schöts 7 [81,82] and Rheinbalme [83]. Lastly, long and slightly curved antler tines showing evidence of use as knapping tools are unique for the Italian peninsula. Antler implements similar to those documented in the Mondeval burial appear in the Balkans during the second half of the VII millennium BCE [84] and are known in the Scandinavian region [58,85,86]. Their presence within the burial goods of Mondeval is totally coherent with the evidence of the use of the punch and pressure techniques for the extraction of laminar and lamellar products documented through the study of lithic items from the burial.

### And death rituals

Lastly, what can we say about the possible social identity of the deceased and the symbolic world of Late Mesolithic groups of the Southern Alps by looking at Mondeval grave goods? Let’s first compare our burial to the funerary evidence of the Alpine area and the Italian peninsula in a diachronic perspective—and then to that of the Late Mesolithic of Southern Europe.

Relating the Mondeval burial with an earlier context in the same area–the Late Epigravettian grave of a young adult male from Riparo Villabruna, dated to 12,140±70 B.P. (KIA-27004) (i.e., 12,237–11,830 BCE) [87] (Fig 1B)–some elements of continuity in funerary traditions can be observed [47,88]. These are evident in the supine position of the body, in the presence of stones on the lower limbs–although those of Villabruna show evidence of painting, which does not seem to be present in Mondeval–and, especially, in the burial furnishing. Also in Villabruna, the latter is composed of a set of varied items–a decorated bone point, a backed knife, a blade, a core, a retoucher, and an agglomerate of resin and propolis–grouped near the left hand of the individual. Similarities can be traced in the location and types of some of the represented objects, as well as in their nature of personal tool-kit of the deceased.

In contrast, a significant gap is documented in the funerary ritual if we compare the burial of Mondeval de Sora to the Mesolithic grave contexts of the Italian peninsula all dated—with few exceptions—to the Preboreal-Boreal period. This gap appears in the poor composition of grave goods of the latter in comparison to the rich burial furnishing of Mondeval de Sora while common traits are found in the generally widespread supine position of the bodies, except for Grotta dell’ Uzzo, and the diffused presence of stone coverings (Fig 1B) [89]. The current data point to a geographic distribution of Italian Mesolithic grave contexts that includes: a) the south-eastern Alps with two adult females from Vatte di Zambana and Borgonuovo di Mezzocorona in the Adige valley (the latter attributed to the Early Mesolithic on the base of stratigraphic observations but with radiocarbon dates supporting an attribution to the Neolithic [90]) with no associated grave goods except for the presence of some traces of ochre; b) central Italy with a debated burial from at Grotta Continenza; in c) Southern Italy with one new-born and one infant discovered at Grotta Praia Mare in Calabria; d) Sicily with eleven burials containing thirteen individuals in total at Grotta dell’Uzzo, three burials at Grotta Molara and one debated Mesolithic burials at Grotta d’Oriente-Oriente B [91]. In all of these contexts, grave goods are either absent or represented by only a few implements, generally belonging to the categories of lithic artifacts, ornamental shells, and bone tools with some gender differences [8]. At Grotta dell’Uzzo, few bone tools are most likely associated with dressing [92]. Lastly, three new burials have recently been discovered in Sardinia at the site of S’Omu and S’orku and dated to the Atlantic chronozone. These attest to a very specific ritual with two individuals accompanied by one large *Charonia lampas* shell [93,94].

Therefore, the search for Late Mesolithic burials that can be compared to the Mondeval de Sora funerary evidence brings us outside the Italian peninsula and, namely, to southern Europe, considering, respectively, the Alps and the western Balkans as the northern and the eastern boundaries of this area.

Across this vast region, multiple burial episodes occur in the same locations during the Late Mesolithic (Moita do Sebastião, Cabeço da Arruda, and Cabeço da Amoreira in the Muge valley, El Collado in Southern Spain, Lepenski Vir, Padina, Schela Cladovei, and Vlasac in the Danube Gorges, Pupicina and Vela Spila in Croatia, Franchthi cave in Greece, Campu Stefanu in Corsica), while others have yielded a lower number of individuals or just one (i.e., Los Canes and La Braña-Arintero in the North of Spain, Poeymau, Montclus and Cuzoul in the South of France) [8,95–100] (Fig 1B). High variability of rituals is documented, especially with regards to the body treatment (primary and secondary inhumations, cremations, excarnation, post-mortem manipulations, single and collective inhumations), body placement (extended supine and laterally flexed being the most diffused positions) [95,101–104] but also of presumed food offers and burial goods, which are sometimes present, but never abundant.

The most recurrent grave goods include ornaments made of marine and freshwater shells, pharyngeal teeth, and stone as well as very few deer teeth common both in female and male burials and often in those of infants [77,103–106]. Osseous and lithic artifacts are less frequent such as in the case of the structure II from Los Canes, Asturia, with a long bone point, a perforated stick, a pebble with pitting traces, and several pierced shells [95,107] (Fig 1B). Ochre is also frequently recorded together with hearths and stones piled on the body. Such a combination of elements, and namely the presence of more than one burial in the same site, is considered by most authors as indicating increased social complexity of these communities. Nonetheless, none of the contexts from this area seems to show any specific elements of comparison with Mondeval [97].

By contrast, considering the number, variety, and arrangement of grave goods, the burial of Mondeval de Sora shares more features with some evidence from the Northern and Eastern European regions [8,108–111]. Different ways of disposing of the dead are documented across this area along with several richly furnished graves accompanied by flint (namely blades) and osseous artifacts and ornaments, the latter being mostly represented by beads made from animal teeth and amber; mass animal graves and ochre are also very frequent Among this rich evidence, the closest comparable context is the grave of Janisławice in Central Poland (Fig 1B) [111]. The deceased individual—a 30-year-old male—was placed in a sitting position with large quantities of ochre. While this specific use of ochre and the position of the deceased may differentiate it from the Mondeval context, there are similarities with other aspects of the assemblage: beads made of animal teeth (here deer and auroch teeth) and, more, a rich set of chert and osseous implements located along the left side of the body. The tool-kit includes bladelet cores and blades/bladelets (some of which were refitted), antler items used in flint-knapping, and several other items made of bone and teeth, which have been interpreted as being part of the tool-kit of the man [112,113]. The grave is dated by ^14^C to 6580±80 B.P. (5645–5375 BCE) [114], therefore around one thousand years later than Mondeval de Sora burial in accordance with the generally more recent age of the Late Mesolithic in this area. The man also seems to display meaningful evidence of an “adaptation to frequently performed tasks” [113], such as the use of a bow and a frequently assumed kneeling or squatting position. The correspondence of the tool-kit as a whole, notably the cores/blades/bladelets together with antler/bone tools, at both Mondeval and Janisławice point to specialized activities performed by these two individuals related to expertise in flint-knapping–which would have defined their identity to such an extent to warrant internment with the body.

## Conclusions

Through the analyses carried out on the rich repertoire of lithic and osseous items accompanying the burial of Mondeval de Sora both daily and funerary habits have been explored, highlighting the effects of the transformations that occurred during the 7th millennium BCE in Western Europe, the origin of which is still debated [4,7]. Namely, our analysis highlighted that the objects located in the burial pit relate to a wide range of activities and thus revealed aspects of technological and economic intensification associated with broader socio-cultural transformations characterizing this period. Their variety and diversified functional histories, along with their location and disposal, seem to strongly reflect the social identity of the individual. Among the resource acquisition practices, some aspects can be emphasized. Firstly, specialization in hunting/fishing which is underlined by the presence of a harpoon, a type of tool which makes its first appearance at the end of the Sauveterrian in the Southern Alps [59], and of a shaft straightener made out of a deer vertebra Secondly the development of a wide-scale lithic raw material provisioning strategy, which is attested by the presence of several cores and blades made from exogenous cherts. In particular, a virtual and symbolic link connects this set of items to at least two antler tines, which were used as punches/pressers, documenting a unique association of this type in a Southern European Late Mesolithic context. Lastly, the processing of vegetal and animal resources aimed at different craftworks is supported by use wear traces developed on almost all the lithic and osseous artifacts. Regarding lithic implements, a particular emphasis concerns laminar blanks, which assume the role of versatile tools to be used either with their rough natural margins for processing soft plant and animal materials or, after modification by retouch, for treating harder substances. While such aspects confirm that plant, antler, and bone processing played a major role in the Late Mesolithic crafting system of the Alpine region, there also seems to have been an intensification in the use of stone tools in plant-working activities, especially those involving siliceous plant processing, towards the end of the Mesolithic in numerous contexts in Northern Europe [74–76,115–118].

Finally, the grave of Mondeval de Sora seems to indicate that the changes which concern the technological and economic systems of the Late Mesolithic societies also affected their funerary sphere [25]. This is suggested by the unusually rich and specialized inventory of items associated with the deceased and the symbolic meaning assumed by some of them. Namely, we suggest that the three blades of unusual dimensions and high technical quality, respectively located under the head and above the shoulders, are indicative of the fact that this man was not only a hunter-fisher highly skilled in vegetal and animal material craftworks but most of all a flint-knapper. The presence of such blades and the privileged location they occupy in the burial ritual could either reflect the status of specialized craftsman of the deceased or, alternatively, constitute a feature of the burial ritual associated to male individuals, as suggested for more recent contexts (Early Ertebolle, Kannegaard 2016; Square Mouthed Pottery (SMP) Neolithic of the Po plain area, [72]. In any case, we argue that the relevance given to these items in the burial ritual could be connected to a renewed symbolic value assumed by laminar blanks in relation to the introduction of the new knapping techniques for laminar extraction. These emerged during the 7th millennium in Southern and Western Europe, and the connection to the appearance, since this age, of specialized craftsmen cannot be excluded on the basis of our results. In this scenario, the evidence from Mondeval de Sora burial certainly highlights the particular position attained by this individual within his society in connection to the special skills he acquired during the lifetime.

## References

[1] Kozlowski SK. Thinking Mesolithic. Oxbow books; 2009.

[2] Clark JGD. Blade and Trapeze Industries of the European Stone Age. Proceedings of the Prehistoric Society. 1958. pp. 24–42.

[3] Marchand G. Beyond the Technological Distinction between the Early and Late Mesolithic. Palethnologie. 2014 10.4000/palethnologie.1107

[4] Perrin T, Marchand G, Allard P, Binder B. Le second Mésolithique d’ Europe occidentale: origines et gradient chronologique. Fond Fyssen-Annales. 2009;24: 163–178.

[5] Hartz S, Terberger T, Zhilin M. New AMS-dates for the Upper Volga Mesolithic and the origin of microblade technology in Europe. Quartär. 2010;57: 155–169.

[6] Biagi P, Starnini E. The Origin and Spread of the Late Mesolithic Blade and Trapeze Industries en Europe: Reconsidering J.G.D. Clark’s Hypothesis Fifty Years After. Interact Chang Meanings Essays honour Igor Manzura occassion his 60th Birthd. 2016; 33–45.

[7] Marchand G, Perrin T. Why this revolution? Explaining the major technical shift in Southwestern Europe during the 7th millennium cal. BC. Quat Int. 2017;428: 73–85. 10.1016/j.quaint.2015.07.059

[8] Grünberg JM. Women and men in Mesolithic burials: inequalities in early postglacial hunter-gatherer-fisher societies. Cetatea de. In: Boroneanţ, A MM, editor. From hunter-gatherers to farmers : human adaptations at the end of Pleistocene and the first part of the Holocene : Papers in Honour of Clive Bonsall. Cetatea de. Târgovişte; 2017. pp. 185–202.

[9] Biagi P. Some Aspects of the Late Mesolithic and Early Neolithic Periods in Northern Italy. From Mesolith to Neolit. 2001;11: 71–88.

[10] Binder D. Mesolithic and Neolithic interaction in southern France and northern Italy: new data and current hypotheses. Eur First Farmers. 2009; 117–143. 10.1017/cbo9780511607851.006

[11] Starnini E, Biagi P, Mazzucco N. The beginning of the Neolithic in the Po Plain (northern Italy): Problems and perspectives. Quat Int. 2018;470: 301–317.

[12] Escalon de Fonton M. Du Paléolithique supérieur au Mésolithique dans le Midi méditerranéen. Bull la Société préhistorique française. 1966;63: 66–180.

[13] Puchol OG, Balaguer LM, Tortosa EAJ, Aubán JB. From the mesolithic to the neolithic: On the mediterranean coast of the Iberian Peninsula. J Anthropol Res. 2009;65: 237–251. 10.3998/jar.0521004.0065.205

[14] Kaczanowska M. The Mesolithic lithic industries of the eastern Adriatic zone. Folia Quat. 2018;86: 191–215. 10.4467/21995923fq.18.003.9821

[15] Collina C. Le Néolithique ancien en Italie du sud. Le Néolithique Anc en Ital du sud. 2020 10.2307/j.ctvxrq0j9

[16] Franco C. La fine del Mesolitico in Italia: identità culturale e distribuzione territoriale degli ultimi cacciatori-raccoglitori. Quaderni—Società per la preistoria e protostoria della Regione Friuli Venezia Giulia; 13. Soc. per la Preistoria e Protostoria della Regione Friuli-Venezia Giulia; 2011.

[17] Fontana F, Ferrari S, Visentin D. A review on the Mesolithic of the Emilian Apennines and Southern Po Plain. Preist Alp. 2013;47: 17–30.

[18] Fontana F, Visentin D. Between the Venetian Alps and the Emilian Apennines (Northern Italy): Highland vs. lowland occupation in the early Mesolithic. Quat Int. 2016;423: 266–278. 10.1016/j.quaint.2015.12.014

[19] Zangrossi F, Delpiano D, Cocilova A, Ferrari F, Balzani M, Peresani M. 3D visual technology applied for the reconstruction of a Paleolithic workshop. Journal of Archaeological Science: Reports. Elsevier Ltd; 2019 10.1016/j.jasrep.2019.102045

[20] Fontana F, Ferrari S. Il Mesolitico in Emilia e il Complesso Culturale Castelnoviano: Dinamiche Insediative e Sistemi Tecnici Litici. Il Mesolitico in Emilia e il Complesso Culturale Castelnoviano: Dinamiche Insediative e Sistemi Tecnici Litici. BAR Publishing; 2016.

[21] Bagolini B, Broglio A. Il ruolo delle Alpi nei tempi preistorici (dal Paleolitico al Calcolitico) Studi di paletnologia in onore di Salvatore M. Puglisi. Universita di Roma La Sapienza; 1985.

[22] Dalmeri G, Pedrotti A. Distribuzione topografica dei siti del Paleolitico superiore finale e Mesolitico in Trentino Alto-Adige e nelle Dolomiti Venete (Italia). Preist Alp. 1994;28: 247–267.

[23] Visentin D, Carrer F, Fontana F, Cavulli F, Cesco Frare P, Mondini C, et al Prehistoric landscapes of the Dolomites: Survey data from the highland territory of Cadore (Belluno Dolomites, Northern Italy). Quat Int. 2016;402: 5–14. 10.1016/j.quaint.2015.10.080

[24] Fontana F. La sepoltura di Mondeval de Sora (Belluno): complessità sociale e modalità insediative degli ultimi cacciatori raccoglitori dell’Italia nord orientale. La Cult del morire nelle Soc Preist e protostoriche Ital. Stud Interdiscip dei dati e loro Tratt informatico dal Paleolit all’Età del Rame. 2006; 269–292.

[25] Fontana F, Flor E, Duches R. Technological continuity and discontinuity in the Romagnano Loc III rock shelter (NE Italy) Mesolithic series. Quat Int. 2016;423: 252–265.

[26] Ferrari S, Fontana F, Mengoli D, Nenzioni G. The Introduction of a New Flaking Technique in the Bologna Plain Area During Late Mesolithic (Castelnovian) and its Relationships with Débitage Processes of Local Flint Raw Materials: Preliminary Considerations. Riv di Sci Preist. 2010;LX: 43–47. 10.1400/204595

[27] Binder D, Collina C, Guilbert R, Perrin T, Garcia-Puchol O. Pressure-knapping blade production in the north-western Mediterranean region during the seventh millennium cal B.C The Emergence of Pressure Blade Making: From Origin to Modern Experimentation. Springer; 2012 pp. 199–217.

[28] Cristiani E, Pedrotti A, Gialanella S. Tradition and innovation between the Mesolithic and Early Neolithic in the Adige Valley (Northeast Italy). New data from a functional and residues analyses of trapezes from Gaban rockshelter. Doc Praehist. 2009;36: 191–205. 10.4312/dp.36.12

[29] Philibert S. Ist vs. IInd Mesolithic in southern France. Functional approach of techno-economic behavior through the Castelnovian of Montclus rock shelter (Gard): First results. Quat Int. 2016;423: 242–251. 10.1016/j.quaint.2016.02.010

[30] Graziosi P. Nuove manifestazioni d’arte mesolitica e neolitica nel riparo Gaban presso Trento. Riv di Sci Preist. 1975;30: 237–278.

[31] Kozlowski SK, Dalmeri G, Con La Collaborazione Di, Bassetti M, Bruhn F, Cusinato A, et al Riparo Gaban: the Mesolithic layers. Preist Alp. 2000;36: 3–68.

[32] Mussi M. Earliest Italy: an overview of the Italian Paleolithic and Mesolithic. Springer Science & Business Media; 2006.

[33] Alciati G, Cattani L, Fontana F, Gerhardinger E, Guerreschi A, Milliken S, et al Mondeval de Sora: a high altitude Mesolithic campsite in the Italian Dolomites. Preist Alp. 1993;28: 351–366.

[34] Fontana F, Guerreschi A, Bertola S, Briois F, Ziggiotti S, others. The Castelnovian burial of Mondeval de Sora (San Vito di Cadore, Belluno, Italy): evidence for changes in the social organisation of Late Mesolithic hunter-gatherers in north-eastern Italy. Mesolithic burials—Rites, symbols and social organisation of early postglacial communities Mesolithische Bestattungen—Riten, Symbole und soziale Organisation früher postglazialer Gemeinschaften. 2016. pp. 741–756.

[35] Fontana F, Vullo N. Organisation et fonction d’un camp de base saisonnier au coeur des Dolomites: le gisement mésolithique de Mondeval de Sora (Belluno, Italie). Les derniers chasseurs-cueilleurs d’Europe occidentale, Actes du colloque international de Besançon, octobre 1998. 2000. pp. 197–208.

[36] Fontana F, Guerreschi A. Highland occupation in the southern Alps during the Early Holocene. Mesolithic on the move: papers presented at the Sixth International Conference on the Mesolithic in Europe, Stockholm 2000. 2003. pp. 96–102.

[37] Bietti A, Boschian G, Crisci GM, Danese E, De Francesco AM, Dini M, et al Inorganic raw materials economy and provenance of chipped industry in some stone age sites of Northern and Central Italy. Coll Antropol. 2004;28: 41–54. 15636064

[38] Peretto C, Biagi P, Boschian G, Broglio A, De Stefani M, Fasani L, et al Living-floors and structures from the lower paleolithic to the Bronze Age in Italy. Coll Antropol. 2004;28: 63–88. 15636066

[39] Fontana F, Govoni L, Guerreschi A, Padoanello S, Siviero A, Thun Hohenstein U, et al L’occupazione sauveterriana di Mondeval de Sora 1, settore I (San Vito di Cadore, Belluno) in bilico tra accampamento residenziale e campo da caccia. Preist Alp. 2009;44: 207–226.

[40] Gerhardinger ME, Guerreschi A. La découverte d’une sépulture mésolithique à Mondeval de Sora (Belluno, Italie). HOMINIDAE Proceedings of the 2nd International Congress of Human Paleontology. 1987. pp. 511–513.

[41] Guerreschi A. Il sito di Mondevàl de Sóra: la sepoltura. Atti del Convegno “Sepolture Preist nelle Dolomiti e primi insediamenti Stor. 1992;19: 89–102.

[42] Alciati G, Pesce Delfino V, Vacca E. Catalogue of Italian Fossil Human Remains from the Palaeolithic to the Mesolithic. Centro Stampa d’Ateneo; 2005.

[43] Alciati G, Coppa A, Macchiarelli R. La dentizione del cacciatore mesolitico di Mondeval de Sora (S. Vito di Cadore, Belluno). Bull di Paletnologia Ital (n.s 4). 1995;86: 197–266.

[44] Alciati G, Pesce Delfino V, Vacca E. Evidenze patologiche rilevate sullo scheletro di Mondevàl de Sora Atti XII Congresso Associazione Antropologica Italiana Antropologia Contemporanea. Palermo, Alia; 1997 pp. 1–3.

[45] Sparacello VS, Villotte S, Shaw C, Fontana F, Mottes E, Starnini E, et al Changing mobility patterns at the Pleistocene-Holocene transition. Paleolit Italy Adv Stud Early Hum Adapt Apennine Penins. 2018; 357–396.

[46] Gazzoni V, Goude G, Dalmeri G, Guerreschi G, Mottes E, Nicolis F, et al Investigating diet of Mesolithic groups in the Southern Alps: an attempt through stable carbon and nitrogen isotope analysese. Bull la Société d’Anthropologie Paris.

[47] Cattani L. Contenuto pollinico di materiali resinosi come elementi di corredo funebre. Antropol Contemp. 1993;16: 55–60.

[48] Bertola S. The flints of Southern Alps (Non Valley, Italy) provenance found in the mesolithic site of Ullafelsen (Sellrain, Tyrol). 2011.

[49] Bertola S. Southern Alpine (Trento Plateau) and Northern Apennine flints: Ages, Distribution and Petrography. Actes la journée la Société préhistorique française Nice, 28–29 mars 2013, Resosources lithiques, Prod Transf entre Alpes Mèditerranee. 2015;5: 55–75.

[50] Van Gijn AL. The Wear and Tear of Flint: Principles of Functional Analysis Applied to Dutch Neolithic Assemblages. Publications of the Institute of Prehistory Y. Leiden; 1990 pp. 51–57.

[51] Rots V. Are Tangs Morphological Adaptations in View of Hafting? Macro- and microscopic wear analysis on a selection of tanged burins from Maisières-Canal. Notae Praehistoricae. 2002;114: 61–69.

[52] Baroni C, Biagi P. Excavations at high altitude Mesolithic site of Laghetti del Crestoso (Bovegno, Brescia—Northern Italy). 1997; 109.

[53] Legrand A. Fabrication et utilisation de l’outillage en matières osseuses du Néolithique de Chypre: Khirokitia et Cap Andreas-Kastros British archaeological Reports—International Series; 1678. British Archaeological Reports Limited; 2007.

[54] D’errico F, Vanhaeren M. Criteria for identifying red deer (Cervus elaphus) age and sex from their canines. Application to the study of Upper Palaeolithic and Mesolithic ornaments. J Archaeol Sci. 2002;29: 211–232. 10.1006/jasc.2001.0687

[55] Broglio A, Cilli C, Giacobini G, Guerreschi A, Malerba G, Villa G. Typological and technological study of prehistoric implements in animal hard tissues. Coll Antropol. 2004;28: 55–61. 15636065

[56] Dalmeri G, Cusinato A, Neri S, Nicolodi F. Le industrie mesolitiche di Riparo Pradestel (Trento). Aspetti tipologici ed evolutivi. Preist Alp. 2008;43: 131–186. Available: http://www.mtsn.tn.it/pubblicazioni/7/43/10_Pradestel.pdf

[57] Broglio A, Kozlowski SK. Tipologia ed evoluzione delle industrie mesolitiche di Romagnano III. Preist Alp. 1984;19: 93–148.

[58] David É, Sørensen M. First insights into the identification of bone and antler tools used in the indirect percussion and pressure techniques during the early postglacial. Quat Int. 2016;423: 123–142.

[59] Cristiani E, Borić D. Appearance and Function of Harpoons in Northeastern Italy In: Gibaja JF, Marreiros J, Mazzucco N, Clemente-Conte I, editors. Hunter-Gatherers’ Tool-Kit A Functional Perspective. Cambridge Scholars Publishing; 2020 p. 338.

[60] Boas F. The Central Eskimo Bureau of American Ethnology. Sixth Annu Report Washingt DC Smithson Inst 1888.

[61] Giddings JL, Rudenko SI, Tolstoy P. The Ancient Culture of the Bering Sea and the Eskimo Problem Ethnohistory. University of Toronto Press; 1962.

[62] Pétillon J-M. What are these barbs for? Preliminary reflections on the function of the Upper Magdalenian barbed weapon tips / Des barbelures pour quoi faire? Réflexions préliminaires sur la fonction des pointes barbelées du Magdalénien supérieur—document. Palethnologie. 2008;1: 66–97.

[63] Rozoy J-G. Les derniers chasseurs: l’Epipaléolithique en France et en Belgique: essai de synthèse. J.-G. Rozoy; 1978.

[64] Owen LR. Distorting the past: Gender and the division of labor in the European Upper Paleolithic. Kerns Verlag; 2005.

[65] St-Pierre CG, Walker RB. Bones as tools: current methods and interpretations in worked bone studies. Archaeopress Oxford; 2007.

[66] David E. Principles of the technological analysis and diagnostic criterias of the Mesolithic techniques Course of the Seminar on Bone technology UPO-MNHN Archives-Ouvertes CEL-SHS du Centre pour la Communication Scientifique Directe CCSd du CNRS, Paris 2016.

[67] Van Gijn AL. The use of bone and antler tools: Two examples from the Late Mesolithic in the Dutch coastal zone Bones as tools: Current Methods and Interpretations in Worked Bone Studies. Archaeopress Oxford; 2007 pp. 81–92.

[68] Van Gijn A, Little A. Tools, use-wear and experimentation: extracting plants from stone and bone. Wild Harvest. Plants hominin pre-agrarian Hum worlds. 2016; 135–153.

[69] Cilli C, Giacobini G, Guerreschi A. Bone artifacts from the Mesolithic burial of Mondeval de Sora (Belluno, North-Eastern Italy) Preliminary technological and functional observations. 3rd International Congress “Science and Technology for safeguard cultural heritage Medit basin”. 2002 pp. 895–899.

[70] Tomasso A, Porraz G. Hunter-gatherer mobility and embedded raw-material procurement strategies in the mediterranean upper paleolithic. Evol Anthropol Issues, News, Rev. 2016;25: 164–174. 10.1002/evan.21488 27312188

[71] Dal Santo N. Sistemi tecnici a confronto: l’evoluzione delle industrie litiche dal Mesolitico recente all’Eneolitico nei siti del medio corso del Panaro. Cardarelli A, Malnati L(a cura di), Atlante dei Beni Archeol della Prov di Modena, III, Alta Pianura e Collina. 2009;1: 36–45.

[72] Bernabò Brea M, Maffi M, Mazzieri P, Salvadei L. Testimonianze funerarie della gente dei Vasi a Bocca Quadrata in Emilia occidentale: archeologia e antropologia. Riv di Sci Preist. 2010;60: 63–126.

[73] Dal Santo N, Mazzieri P. Il sito di VBQ iniziale di Ponte Ghiara (Parma): le industrie litiche e ceramiche. Origini. 2010;32: 105–160.

[74] Gassin B, Marchand G, Claud É, Guéret C, Philibert S. Les lames à coches du second Mésolithique: Des outils dédiés au travail des plantes? Bull la Soc Prehist Fr. 2013;110: 25–46. 10.3406/bspf.2013.14227

[75] Beugnier V, Crombé P. Plant processing from a prehistoric and ethnographic perspective. BAR international series; 1718. John & Erica Hedges Ltd.; 2007.

[76] Little A, Van Gijn A. Enigmatic plant-working tools and the transition to farming in the Rhine/Meuse Delta. Analecta Praehist Leiden. 2017;47: 1–9.

[77] Cristiani E, Borić D. Mesolithic harpoons from Odmut, Montenegro: Chronological, contextual, and techno-functional analyses. Quat Int. 2016;423: 166–192. 10.1016/j.quaint.2015.11.010

[78] Clark R. The Mesolithic Hunters of the Trentino: A Case Study in Hunter-Gatherer Settlement and Subsistence from Northern Italy The Mesolithic Hunters of the Trentino: A Case Study in Hunter-Gatherer Settlement and Subsistence from Northern Italy. British Archaeological Reports Ltd; 2000 10.30861/9781841711256

[79] Wierer U, Betti L, Boscato P, Bazzanella M, Boschin F, Crezzini J, et al. Living near the water. Environment, wetland economy and fishing techniques of the Mesolithic site Galgenbühel/Dos de la Forca in the Adige Valley (South Tyrol, Italy). Proceedings of the Conference, Besançon. 2013.

[80] Dalmeri G, Grimaldi S, Lanzinger M. Il Paleolitico e il Mesolitico. Stor del Trentino. 2001;1: 15–118.

[81] Nielsen EH. Paläolithikum und Mesolithikum in der Zentralschweiz, Mensch und Umwelt zwischen 17000 und 5500 vor Christus. Luzern, Kantonsarchäologie Archäologische Schtriften Luzern; 2009 p. 720.

[82] Nielsen EH. The Mesolithic background for the Neolithisation process. Doc Praehist. 2009;36: 151–158. 10.4312/dp.36.9

[83] Laus S. Rheinbalme-Krinnenbalme: zwei steinzeitliche Abri-Stationen bei Koblach in Vorarlberg; ein Beitrag zur Erforschung der sozioökonomischen Strukturen bei Wildbeutern und frühen Bauern im Alpenrheintal. na; 2006.

[84] Borić D, Borovinić N, Duričić L, Bulatović J, Gerometta K, Filipović D, et al Spearheading into the Neolithic: Last Foragers and First Farmers in the Dinaric Alps of Montenegro. Eur J Archaeol. 2019;22: 470–498. 10.1017/eaa.2019.14

[85] Karsten P, Knarrström B. Tågerup—Unearthing a Mesolithic Society. BAR Intern. In: Crombé P, editor. Acts of XIVth UISPP Congress, University of Liège, Belgium, 2–8 September 2001 Section 7: the Mesolithic. BAR Intern. Oxford: Archaeopress.; 2004. pp. 39–46.

[86] Sørensen M. Teknologiske traditioner i Maglemosekulturen. En diakron analyse af Maglemosekulturens flækkeindustri. Stenalderstudier. Jysk Arkæologisk Selskab; 2006. pp. 19–76.

[87] Vercellotti G, Alciati G, Richards MP, Formicola V. The Late Upper Paleolithic skeleton Villabruna 1 (Italy): a source of data on biology and behavior of a 14.000 year-old hunter. J Anthr Sci. 2008;86: 143–163. 19934473

[88] Broglio A. Les sépultures épigravettiennes de la Vénétie (abri Tagliente et abri Villabruna). Nat Cult. 1995; 845–867.

[89] Gazzoni V, Fontana F. Quelle mort? Quelle vie? Pratiques funéraires et organisation sociale des chasseurs-cueilleurs de la péninsule italienne. Bull Mem Soc Anthropol Paris. 2011;23: 52–69.

[90] Dalmeri G, Mottes E, Nicolis F. La sepoltura mesolitica di Mezzocorona-Borgonuovo (Tn). Atti della XXXIII Riun Sci dell’Istituto Ital di Preist e Protostoria, Trento 1997. 2002; 21–24.

[91] Martini F. Sepolture e rituali funerari del Mesolitico in Italia. In: Martini F, editor. La cultura del morire nelle società preistoriche e protostoriche italiane Studio interdisciplinare dei dati e loro trattamento informatico. Origines, Progetti; 2006. pp. 67–86.

[92] Collina C. Gli oggetti di corredo nelle sepolture mesolitiche della Grotta dell’Uzzo (Trapani): studio tecnologico e analisi delle tracce. La Cult del morire nelle Soc Preist e protostoriche Ital Stud Interdiscip dei dati e loro Tratt informatico dal Paleolit all’età del Rame. 2006; 239–268.

[93] Floris R, Melis RT, Mussi M, Palombo MR, Iacumin P. La presenza umana nella Sardegna centro occidentale durante l’Olocene antico: il sito di S’Omu e S’Orku, Arbus, VS. Atti della XLIV Riun Sci la Preist e la protostoria della Sardegna Cagliari, Barumini, Sassari 23–28 novembre 2009, vol 2–3 Comun vol 4 Posters. 2012; 999–1004.

[94] Melis RT, Mussi M. Mesolithic burials at S’Omu e S’Orku (SOMK) on the south-western coast of Sardinia. Mseolithic burials—Rites, Symb Soc Organ early Postglacial Communities (International Conf Halle, Ger 18th-21st Sept 2013). 2016; 733–740.

[95] Arias Cabal P, Alvarez Fernández E. Les chasseurs-cueilleurs de la Péninsule ibérique face à la mort : une révision des données sur les contextes funéraires du Paléolithique supérieur et du Mésolithique. La spiritualité actes du Colloq Int Liège (10–12 décembre 2003). 2004; 221–236.

[96] Vidal Encinas JM, Prada Marcos ME, Fernández Rodríguez C, Fuertes Prieto MN, Fuertes Prieto N, Fernández Rodríguez C. Los hombres mesolíticos de la Braña-Arintero (Valdelugeros, León): el hallazgo, situación, aspectos arqueo-antropológicos, cronología y contexto cultural. Férvedes. Congreso internacional de arqueología de Vilalba. Universidad de León. 2010.

[97] Arias P. Grave goods in the Mesolithic of Southern Europe: an overview. Mesolith burials—Rites, Symb Soc Organ early postglacial communities. 2016; 693–704.

[98] Terradas X, Gibaja J, Subirà M, Santos F, Agulló L, Gómez-Martínez I, et al The Mesolithic cemetery of El Collado. State of the art and new results In: Grünberg J., Gramsch B., Larsson L., Orschiedt J. and H M, editor. Mesolithic burials—Rites, symbols and social organisation of early postglacial communities Band II. Halle; 2016 pp. 705–718.

[99] Verjux C. Les pratiques funéraires mésolithiques en Europe. Diversité dans l’espace et dans le temps. Art, archéologie Patrim. 2007; 15–35.

[100] Courtaud P, Petersen HC, Zemour A, Leandri F, Cesari J. The Mesolithic burial of Campu Stefanu (Corsica, France) Mesolithic Burials-Rites, symbols and social organisation of early postglacial communities. 2016 pp. 719–731.

[101] Cullen T. Mesolithic mortuary ritual at Franchthi Cave, Greece. Antiquity. 1995;69: 270–289. 10.1017/S0003598X00064681

[102] Schulting RJ. Creativity’s coffin: Innovation in the burial record of mesolithic Europe. Creat Hum Evol Prehistory. 2005; 148–165.

[103] Boroneant A, Bonsall C. Burial practices in the Iron Gates Mesolithic. Proceedings of the International Symposium on Funerary Anthropology. 2012. pp. 45–56.

[104] Borić D, French CAI, Stefanović S, Dimitrijević V, Cristiani E, Gurova M, et al Late Mesolithic lifeways and deathways at Vlasac (Serbia). J F Archaeol. 2014;39: 4–31. 10.1179/0093469013Z.00000000070

[105] Cristiani E, Borić D. 8500-year-old Late Mesolithic garment embroidery from Vlasac (Serbia): Technological, use-wear and residue analyses. J Archaeol Sci. 2012;39: 3450–3469. 10.1016/j.jas.2012.05.016

[106] Borić D, Cristiani E. Special Issue: Personal Ornaments in Early Prehistory Taking Beads Seriously: Prehistoric Forager Ornamental Traditions in Southeastern Europe. PaleoAnthropology. 2019;208: 239.

[107] Tresguerres J. Azilian Burial from Los Azules I, Asturias, Spain. Curr Anthropol. 1976;17: 769–770. 10.1086/201830

[108] Pequart M, Pequart S-J. Hoëdic. Deuxieme station-necropole du Mesolithique cotier Armorican. de Sikkel; 1954.

[109] Péquart M, Péquart S-J, Boule M, Vallois HV. Téviec, Station-nécropole Mésolithique du Morbihan. Archives de l’Institut de Paléontologie humaine; 18. Masson et Cie; 1937.

[110] Brzozowski J, Siemaszko J. Ochre and beads. The hunter’s style of the burials in the Polish Mesolithic,[w:] Actes du Symposium International Préhistoire des Pratiques mortuaires Paléolithique-Mésolitique-Néolitique. 1999.

[111] Sulgostowska Z. New data concerning Mesolithic burials in Polish territory. Tagungen d. In: J. Grünberg, B. Gramsch, L. Larsson, J. Orschiedt and HM, editor. Mesolithic burials–Rites, symbols and social organisation of early Postglacial communities. Tagungen d. Halle; 2016. pp. 439–455.

[112] Barska, K., W. Migal, A. Gręzak, K. Pyżewicz, M. Wąs and WG 2015. Janisławice Man–A reinterpretation of the Mesolithic grave. In: Borić D., editor. Ninth International Conference on the Mesolithic in Europe. Belgrade; 2015.

[113] Stanaszek ŁM, Mańkowska-Pliszka H. A New osteological analysis of Janisławice Man. Tagungen des Lanndesmuseums für Vor Halle. 2015;13: 1–8.

[114] Cyrek M, Cyrek K. La sépulture mésolithique de Janisławice. L’Institut d’Histoire de la Culture Materielle de L’Académie Polonaise des Sciences; 1980.

[115] Jensen HJ. Flint tools and plant working: hidden traces of Stone Age technology: a use wear study of some Danish Mesolithic and TRB implements. Choice Reviews Online. Aarhus Universitetsforlag; 1994.

[116] Hurcombe LM. Plant processing for cordage and textiles using serrated flint edges: new chaînes opératoires suggested by combining ethnographic, archaeological and experimental evidence for bast fibre processing. Plant Processing from a Prehistoric and Ethnographic Perspective Proceedings of a workshop at Ghent University (Belgium) November 28, 2006. 2007. pp. 41–66.

[117] Guéret C. Character and variability of Early Mesolithic tool-kits in Belgium and Northern France: The contribution of a functional approach. Mesolith Palethnography Res open-air sites between Loire Neckar Proc from Int round-table Meet Paris, Novembre 26–27, 2010. 2013; 147–167.

[118] Bergsvik KA, David É. Crafting Bone Tools in Mesolithic Norway: A Regional Eastern-Related Know-How. Eur J Archaeol. 2015;18: 190–221. 10.1179/1461957114y.0000000073

